# Natural Products for Improving Soft Tissue Healing: Mechanisms, Innovations, and Clinical Potential

**DOI:** 10.3390/pharmaceutics17060758

**Published:** 2025-06-08

**Authors:** Adina Alberts, Ioana Alexandra Lungescu, Adelina-Gabriela Niculescu, Alexandru Mihai Grumezescu

**Affiliations:** 1 Carol Davila University of Medicine and Pharmacy, 050474 Bucharest, Romania; adina-magdalena.alberts@rez.umfcd.ro; 2 National University of Science and Technology Politehnica Bucharest, 011061 Bucharest, Romania; alexandralungescu@gmail.com (I.A.L.); agrumezescu@upb.ro (A.M.G.); 3 Research Institute of the University of Bucharest—ICUB, University of Bucharest, 050657 Bucharest, Romania

**Keywords:** natural products, scar-free healing, wound healing, anti-inflammatory, antioxidant, fibrosis inhibition, bioactive peptides, nanotechnology, bioavailability, regenerative medicine

## Abstract

Scar development is a notable clinical and aesthetic issue in soft tissue healing, frequently compromising functionality and quality of life. Conventional treatments demonstrate limited efficacy in avoiding fibrosis and facilitating regenerative repair. Nevertheless, natural compounds have surfaced as viable alternatives owing to their biocompatibility, multitarget bioactivity, and historical application in traditional medicine. This review examines the therapeutic potential of plant-derived substances, marine agents, and microbial metabolites in influencing critical stages of wound healing, including inflammation, oxidative stress, fibroblast activation, and extracellular matrix remodeling. While these agents have demonstrated beneficial effects in preclinical models, their direct impact on functional or aesthetic clinical outcomes remains under investigation. We propose a hierarchical framework linking molecular mechanisms to clinical endpoints, suggesting that improvements at the cellular and molecular level may eventually support better healing quality. Natural bioactives, especially when integrated into advanced delivery systems such as hydrogels and nanocarriers, show promise for enhancing the regenerative microenvironment. By contextualizing these mechanisms within real-world therapeutic goals, this review highlights both the potential and limitations of natural products in the pursuit of improved soft tissue healing. Further translational research is needed to determine how modulation of these processes may reduce scarring and approach clinically meaningful outcomes.

## 1. Introduction

**Figure 1:**
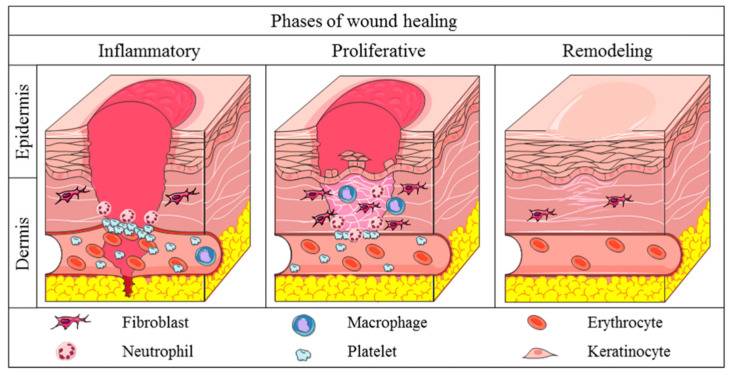
The three main stages of wound healing. Reprinted from an open-access source [3].

Cutaneous wound healing is a crucial physiological process involving the collaboration of several cell types and their byproducts [1,2]. The wound healing process has four interconnected and overlapping phases: hemostasis, inflammation, proliferation, and tissue remodeling or resolution, as shown in Figure 1 [3]. The phases and their biophysiological activities must transpire in the correct order, at a designated time, and persist for a certain length at an appropriate intensity. Numerous variables may influence wound healing, disrupting one or more steps of the process and resulting in inadequate or impaired tissue restoration [4,5].

The process encompasses a sequence of synchronized cellular and molecular activities that advance through four primary phases: hemostasis, inflammation, proliferation, and remodeling. Every step is essential for the restoration of tissue architecture and functionality. Table 1 presents a comprehensive summary of these stages, including the main cellular and biophysiological events linked to each stage and a succinct discussion of the fundamental mechanisms involved. This systematic method emphasizes the transition from early clot formation to tissue remodeling, demonstrating the complex processes that enable efficient wound healing.

Fibroblasts function as key regulators in physiological wound healing, involving extracellular matrix (ECM) deposition, remodeling, and wound contraction, promoting tissue repair and restoring tissue integrity. The deregulation of fibroblast activity results in hypertrophic scars and keloids, marked by excessive collagen accumulation and abnormal ECM remodeling. Fibroblasts in scar tissue display phenotypic alterations characterized by enhanced proliferation, collagen production, and differentiation into myofibroblasts, hence sustaining the fibrotic phenotype [12,13]. Various molecular mediators, including TGF-β, PDGF, and FGF, tightly govern fibroblast activity and role in scar formation. Thus, comprehending the intricate interaction between fibroblasts and the microenvironment is crucial for clarifying the processes promoting scar formation and pinpointing possible treatment targets [4].

Scarring is an inherent result of the healing process, which, nonetheless, can result in considerable physical, aesthetic, and emotional difficulties, significantly affecting an individual’s overall health and quality of life. Understanding these challenges is essential for developing strategies to improve scar management and patient care [8,14]. Scars, especially those arising from significant burns, can limit mobility when they develop close to joints or regions that experience regular movement. This constraint stems from an overabundance of collagen accumulation and scarring, leading to diminished tissue pliability. Contracture scars, frequently seen in individuals with burn injuries, can result in persistent mobility challenges and may necessitate physical therapy or surgical procedures. In many cases of thermal injuries, up to 70% of individuals develop hypertrophic scars, which greatly exacerbate functional limitations and impede the ability to resume daily activities [13,15,16].

Compared to normal skin, scar tissue is characterized by lower flexibility and moisture, leading to stiffness, soreness, and itching symptoms that may last for many months or even years after the injury, leading to pain and functional difficulties. Scars, especially hypertrophic or keloid scars, may cause neuropathic pain because they include nerves that have been trapped within fibrous tissue. It has been shown via research that severe proliferative scarring may affect the quality of life of burn victims, hence stressing the need to develop appropriate treatments [8]. Individuals who have noticeable scars may be subjected to prejudice in societies that place a high value on superficial appearances. The cultural beliefs of scars may have a role in social isolation and hesitation, negatively impacting overall well-being. Patients who have suffered burns often struggle to adjust to their changed appearance, which may make it difficult for them to reintegrate into society and maintain intimate relationships [14,15].

The modulation of wound healing and fibrosis through natural compounds has garnered significant attention due to their bioactive properties. These compounds, derived from plants, marine organisms, and bacteria, demonstrate the potential to reduce inflammation, counter oxidative stress, and inhibit fibrosis, thereby minimizing excessive scar formation and promoting regenerative repair. Table 2 summarizes key mechanisms involved in wound healing and fibrosis, highlighting the associated problems and the natural compounds that have shown efficacy in regulating these processes. By targeting pathways related to inflammation, oxidative stress, collagen accumulation, and angiogenesis, these natural compounds hold promise for therapeutic applications in wound management [17].

Compounds including curcumin, flavonoids, terpenoids, and polysaccharides have shown efficacy in reducing oxidative stress, inhibiting profibrotic signaling pathways, and promoting angiogenesis without inducing fibrosis. These characteristics render natural products a significant strategy in regenerative medicine, providing potential for non-invasive, biocompatible therapies focused on facilitating scar-free healing [7].

**Figure 2:**
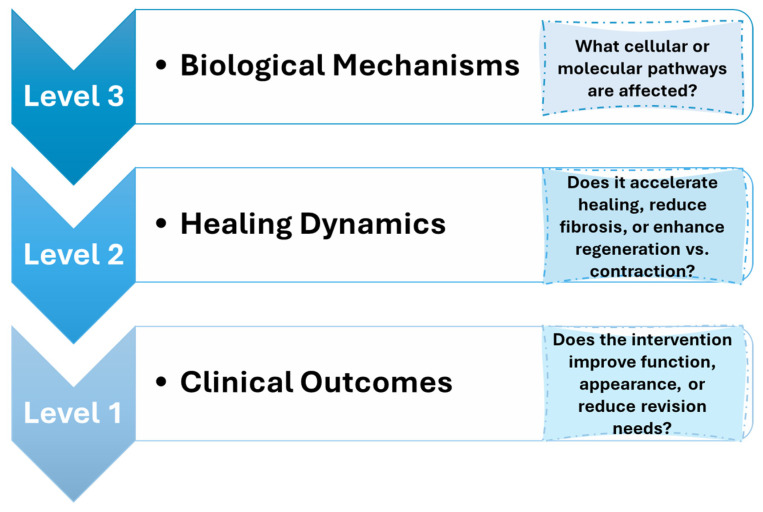
Conceptual framework linking biological mechanisms to clinical outcomes in soft tissue healing.

In this context, this review aims to provide a comprehensive analysis of the therapeutic potential of natural compounds in modulating wound healing and fibrosis. This study explores the bioactive compounds derived from various sources, including plants, marine organisms, and microorganisms. These compounds play roles in alleviating inflammation, reducing oxidative stress, influencing collagen synthesis, and regulating angiogenesis. Figure 2 illustrates a hierarchical framework connecting these biological mechanisms (Level 3) to healing dynamics such as regeneration versus contraction (Level 2) and ultimately to clinical outcomes (Level 1), including functional recovery, aesthetic appearance, and the need for surgical revision. By situating molecular findings within this broader context, the review highlights how natural agents may contribute to improved healing outcomes and underscores the importance of bridging mechanistic insights with patient-centered goals. This integrated perspective supports the growing interest in natural and minimally invasive alternatives to conventional wound care strategies.

While the specific clinical thresholds for “acceptable” healing—such as what constitutes minimal scarring or optimal functional recovery—are application-dependent and not yet fully established, this review discusses the biological mechanisms (Level 3) and their proposed impact on healing outcomes (Levels 2 and 1), particularly in the context of emerging innovations and clinical case studies.

The following sections are structured according to the framework displayed in Figure 2. Section 2, Section 3, Section 4 and Section 5 examine molecular mechanisms of fibrosis and healing modulation. Section 6 introduces emerging material-based delivery systems that aim to improve healing dynamics. Section 7 connects these findings to specific clinical applications—including burns, diabetic ulcers, and surgical wounds—where the potential for improved outcomes can be assessed. Section 8 and Section 9 address translational limitations and future directions needed to bridge the gap between biological modulation and clinically meaningful tissue regeneration.

## 2. The Biology of Scar Formation

This section reviews the cellular and molecular mechanisms underlying scar formation (Level 3). Mechanisms like excessive fibroblast activation, disrupted collagen alignment, and sustained oxidative stress influence healing dynamics such as the balance between regeneration and contraction (Level 2), which ultimately affect functional and aesthetic outcomes (Level 1). However, the extent to which modulating a specific biological process leads to measurable improvements in clinical outcomes remains an open area of research. These relationships are often application-dependent—for instance, acceptable scar stiffness or pigmentation may vary between surgical, burn, or diabetic ulcer settings.

### 2.1. Extracellular Matrix

ECM is an intricate, evolving, and meticulously structured network of macromolecules that is essential for preserving tissue integrity, modulating cellular activities, and coordinating the wound healing process. ECM is now recognized as a dynamic influencer of cellular activities, affecting adhesion, movement, growth, specialization, and programmed cell death through various biochemical and mechanical signaling mechanisms. In addition to offering mechanical support, it serves as a reservoir for growth factors and regulates hydration levels, pH balance, and cellular interactions within the tissue microenvironment [18,19,20].

The most abundant protein in the ECM is collagen, which offers tensile strength and structural stability. Elastin is another important component that contributes to elasticity, enabling tissues to return to their original shape after experiencing mechanical stress. Fibronectin, laminins, and vitronectin are crucial for cell adhesion and migration, significantly contributing to wound repair. Proteoglycans like hyaluronan, decorin, versican, and perlecan bind water molecules and growth factors, influencing extracellular hydration and biomechanical characteristics [9]. Glycoproteins such as integrins facilitate interactions between cells and the extracellular matrix, whereas components of the basement membrane, including collagen IV, laminins, and nidogens, offer specialized support to epithelial and endothelial cells. MMPs are involved in the remodeling of the ECM, allowing it to consistently adjust to physiological and pathological states [21].

The ECM experiences a meticulously controlled transformation to support tissue restoration throughout the wound healing process. At the outset, fibrin and fibronectin create a temporary matrix that reinforces the wound and offers a framework for cell movement. Platelets emit signals that attract immune cells, removing debris and releasing cytokines that stimulate fibroblast activity. Fibroblasts contribute to the deposition of extracellular matrix components, mainly collagen and proteoglycans, which are essential for forming granulation tissue [21]. As time progresses, type III collagen is slowly substituted by type I, enhancing tensile strength. At the same time, the growth factors embedded in the extracellular matrix promote angiogenesis, guaranteeing the supply of oxygen and nutrients to the tissue undergoing regeneration. Ultimately, crosslinking and the breakdown of surplus extracellular matrix enhance the newly developed tissue, reinstating its functionality and averting excessive scarring [9,22].

Excessive ECM deposition leads to fibrosis and scarring, which impairs tissue function, whereas inadequate extracellular matrix formation causes chronic wounds and slows the healing process. The extracellular matrix is essential for sustaining stem cell environments, affecting lineage determination and the capacity for regeneration. The equilibrium between structural strength and biochemical communication renders the extracellular matrix vital for successful wound healing, facilitating synchronized tissue restoration and enduring functionality [18]. Excessive or misaligned collagen deposition contributes to rigid, raised scars. While ECM remodeling is a Level 3 process, its modulation is believed to affect tissue pliability and appearance—key Level 1 outcomes. However, the degree of collagen normalization required to achieve clinically acceptable function or cosmesis is not yet fully established [23,24]. Grasping its complex regulatory systems offers essential perspectives for treatment approaches to enhance regenerative therapies and refine tissue restoration.

### 2.2. Cellular and Molecular Players

Fibroblasts are crucial mesenchymal cells in wound healing that facilitate tissue regeneration by manufacturing and rebuilding the extracellular matrix, modulating inflammation, and engaging with immune and epithelial cells [25,26]. In response to damage, inflammatory cytokines, including IL-1, IL-6, and TNF-α, activate and proliferate fibroblasts, therefore commencing their essential function in the healing process. These cells participate in the development of granulation tissue and release MMPs, promoting immune cell infiltration and ECM remodeling [25,27].

A vital group of fibroblasts, termed myofibroblasts, arises during the proliferative phase and is mostly accountable for wound contraction via the production of α-smooth muscle actin (α-SMA) [28,29]. Under typical physiological settings, myofibroblasts experience apoptosis after tissue healing. When the resolution phase is dysregulated—due to chronic inflammation, mechanical stress, or abnormal signaling, myofibroblasts may remain in the wound environment, resulting in excessive collagen deposition and the formation of hypertrophic scars or fibrotic lesions [21].

The development of fibrosis is also affected by the interaction between fibroblasts and macrophages. The prompt shift from proinflammatory (M1) to anti-inflammatory (M2) macrophages is essential for attenuating inflammation and facilitating tissue reorganization [21,27,30]. M2 macrophages release substances, including transforming TGF-β, which, while essential for extracellular matrix formation and fibroblast activation, may also lead to fibrosis if generated in excess [27].

Moreover, keratinocytes engage with immune cells and fibroblasts via cytokines and chemokines, significantly contributing to the restoration of epidermal integrity [27,31,32]. Imbalances in these cellular processes may extend inflammation, promote myofibroblast viability, and elevate the likelihood of pathological scarring [20,31,33]. Consequently, understanding the control of fibroblast activation and resolution is essential for formulating therapeutic methods to reduce fibrosis and enhance wound healing outcomes [30,32].

### 2.3. The Influence of Cytokines in Fibrosis

TGF-β is a cytokine that regulates ECM production and cellular differentiation. TGF-β signaling is a significant driver of fibrosis, with its increased activity linked to fibrotic development in multiple organs [34,35]. Fibrosis typically involves the conversion of fibroblasts into myofibroblasts, including cancer-associated fibroblasts (CAFs). Various cytokines and chemokines (miR-214, IL-1, α-SMA, integrin β-1) and signaling pathways (EGFR, Wnt/β-catenin, Hippo, TGF-β, JAK/STAT cascades) convert normal fibroblasts into CAFs. The mechanisms underlying CAF transformation are not well understood [36].

**Figure 3:**
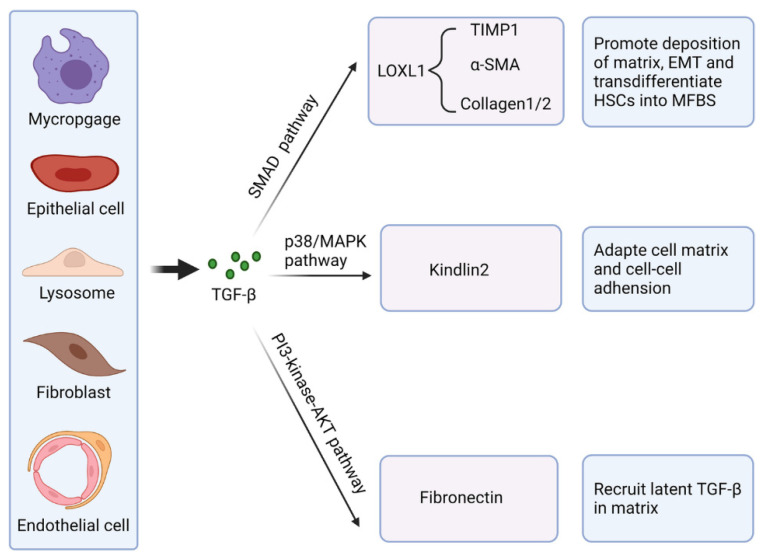
Essential functions of TGF-β in fibrosis. Reprinted from an open-access source [34].

TGF-β I and III exhibit fibrogenic effects and possess 70–82% amino acid homology. TGF-β I is recognized as the principal factor in liver, kidney, and lung fibrosis via canonical and non-canonical signaling pathways. TGF-β is typically upregulated during tissue injury, inflammation, and wound healing. The essential functions of TGF-β in fibrosis are represented in Figure 3 [34]. The prolonged contractile state of the wound enhances ECM protein expression. Dysregulated TGF-β signaling facilitates pathological fibrosis and tumorigenesis through excessive ECM deposition. The abnormal accumulation of ECM initiates fibrosis and immunosuppression by connecting SMAD4, BRAF, and TP53 mutations with MYC amplification, thereby contributing to the CAF phenotype. Inhibition of TGF-β signaling and its downstream pathways significantly reduces fibrosis [35].

### 2.4. Role of Oxidative Stress

Wound healing entails the continuous formation of new blood vessels, cell proliferation, and reorganization of the wound tissue [37]. Recent evidence indicates that ROS significantly influence the physiological and pathological aspects of wound healing. ROS play a role in skin tissue regeneration by regulating inflammation, cell proliferation, angiogenesis, granulation, and extracellular matrix formation. These species are highly active and oxidizing compounds, including the superoxide anion (O^2−^), hydrogen peroxide (H_2_O_2_), and hydroxyl radical (^−^OH). Under normal physiological conditions, moderate ROS levels contribute to cellular signaling and immune responses [38]. External factors like injury, inflammation, or radiation exposure significantly increase ROS production, leading to oxidative stress and damage to skin tissues. Excessive production and accumulation of ROS surpassing cellular defense mechanisms hinder the transition of wound tissue from the inflammatory to the proliferative stage. As a result, the wound area experiences chronic inflammation, leading to delayed healing. Maintaining REDOX homeostasis in cells can prevent abnormal cell growth and immune dysregulation [37].

Persistent oxidative stress exacerbates inflammation and fibroblast overactivity, both of which can lead to fibrosis. Antioxidant modulation may improve healing dynamics (Level 2), but its direct correlation to reduced need for scar revision (Level 1) has not been clearly quantified in clinical settings. Nonetheless, emerging research indicates that antioxidants may accelerate wound healing, particularly in chronic cases [37].

## 3. Natural Products in Soft Tissue Healing

### 3.1. Historical and Traditional Uses

Herbal remedies have played a crucial role in traditional healing practices for centuries, offering natural solutions for various ailments, including the treatment of wounds. Leveraging the healing properties of plants, resins, and botanical extracts, these treatments had been used to reduce inflammation, prevent infection, and promote tissue regeneration long before modern medicine came into play. The long-standing and widespread application of herbal treatments in historical wound care reflects deep empirical knowledge and cultural practices that still influence contemporary natural healing approaches [39]. Thus, through practical observations, numerous natural solutions have been long used to improve soft tissue regeneration at Levels 1 (clinical outcomes) and 2 (healing dynamics), despite not fully understanding the biological processes (Level 3) behind the obtained results.

**Figure 4:**
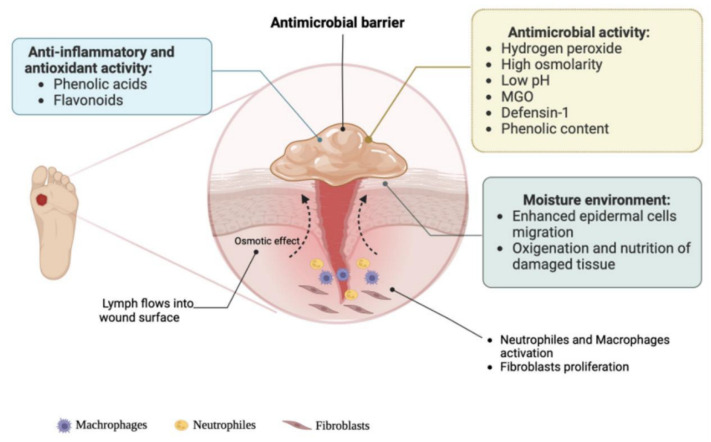
Mechanism of wound healing for honey. Reprinted from an open-access source [41].

One of the most widely historically utilized natural substances for wound treatment is honey. This natural product has been used for 2000 years in wound treatment, and while it has recently regained favor, it may still lack the respect it merits [40,41]. Honey may be a beneficial supplement to the existing array of wound care treatments because of its properties: antimicrobial characteristics, capacity for autolytic debridement and deodorization, anti-inflammatory characteristics, capability to promote tissue regeneration, proficiency in pain management, and scar reduction. As seen in Figure 4 [41], honey possesses anti-inflammatory, antioxidant, and antibacterial properties, making it a natural substance often cited in traditional medicine and increasingly studied for chronic wound treatment [40,42]. Current evidence indicates that honey’s composition and antimicrobial properties are influenced by geographic conditions, hive environment, bee metabolic activity, and processing and storage conditions, resulting in varying characteristics and effectiveness against different microbial strains. Manuka honey, sourced from the nectar of the *Lepstospermum scoparium* plant in New Zealand, is noted for its elevated levels of methylglyoxal (MGO), which provide significant antibacterial properties. Polyphenols significantly influence a honey’s color, taste, and antioxidant and antimicrobial properties, contingent upon its floral sources. Despite variations in geographic and botanical origins, nearly all honey types exhibit bactericidal activity, highlighting their potential for drug development [43,44,45].

*Panax ginseng*, known as Asian ginseng, has been esteemed for thousands of years in East Asian medical practices as a comprehensive healing tonic. Traditional Chinese medicine (TCM) has recognized ginseng for over 2000 years as a strengthening herb that is thought to enhance the body’s qi (vital energy) and facilitate recovery from illness or injury. Classical Chinese texts do not highlight *Panax ginseng* as a direct wound dressing; however, its reputation as a “cure for all ailments” led to its internal use for strengthening weakened patients and promoting healing. *Panax notoginseng*, known as Tienchi ginseng or “Sanqi,” gained prominence in 16th-century China for its applications in trauma care. Herbalist Li Shizhen [46] noted that Sanqi was “the most important military medicine for external injuries from swords, knives, and arrows,” leading to its nickname “the mountain herb that glues wounds together like lacquer.” Historically, soldiers used powdered *Panax notoginseng* root to halt bleeding and facilitate wound closure during battle [47]. Outside of China, American ginseng (*Panax quinquefolius*) was utilized minimally by Native Americans; however, a significant ethnobotanical account reveals that Seminole healers in Florida employed it for the treatment of gunshot wounds. Historical accounts demonstrate that ginseng roots were esteemed across cultures for their healing properties, serving as internal restoratives or, in the case of *Panax notoginseng*, as topical treatments for wounds, well before modern pharmacology recognized their benefits [46,48].

Chamomile, including German chamomile (*Matricaria chamomilla*) and Roman chamomile (*Chamaemelum nobile*), has a longstanding history in Eurasian folk medicine, particularly for treating skin and wound conditions. Historical records indicate that chamomile was utilized in ancient Egypt, Greece, and Rome for its calming and therapeutic effects. The Egyptians revered chamomile, associating it with their sun god, and used crushed flowers on the skin, believing it could heal various ailments, such as wounds and inflammations. Classical Greek physicians like Dioscorides and Pliny the Elder documented chamomile’s efficacy in treating sores, ulcers, and wounds, typically using the flowers in poultices or washes to alleviate swelling [49]. During medieval Europe, chamomile emerged as a key component in herbal wound treatments, notably cited as one of the nine sacred healing herbs in an Anglo-Saxon leech book, utilized in poultices and “herb baths” for injuries. Traditional methods involved steeping chamomile blossoms to create infusions or compresses applied to cuts, abscesses, and burns for cleansing and promoting healing. Chamomile’s mild antiseptic and anti-inflammatory properties have long been acknowledged; its topical applications, including lotions, powdered herbs, and salves, have been utilized for centuries in folk medicine to address skin diseases and wounds. European healers utilized chamomile tea to cleanse wounds and applied dressings infused with chamomile oil to alleviate pain and encourage granulation. The persistent application of chamomile in various cultures underscores its significance as a mild yet potent traditional herb for wound healing, utilized from ancient battlefield treatments to rural folk remedies [49].

Turpentine oil, derived from pine resin, along with similar pine resin products, has a longstanding history as an antiseptic treatment for wounds in both Eastern and Western medical practices. During the Hippocratic era in the ancient Mediterranean (5th–4th century BCE), physicians utilized pine derivatives for the treatment of wounds. The Hippocratic text On Wounds details a pine-based emollient plaster used externally to close wounds, utilizing the resin’s astringent and anti-inflammatory properties. Greek and Roman physicians incorporated pine resin or pitch into salves and poultices for the treatment of ulcers, lacerations, and gangrenous sores, observing that these applications facilitated the cicatrization of serious wounds. Dioscorides in the 1st century CE noted that powdered pine bark and resin could be used on abrasions, infected wounds, and burns to cleanse and promote healing. These practices persisted throughout the European Middle Ages and Renaissance. An illustrative case is the 16th-century French surgeon Ambroise Paré [48], who identified a more humane method for treating gunshot wounds with a salve composed of egg yolk, rose oil, and turpentine. Paré [48] noted that wounds in soldiers treated with a turpentine-based ointment exhibited reduced pain and inflammation compared to those cauterized with boiling oil, prompting him to advocate for the salve as a new standard for battlefield wound care. Pine resin, derived from *Pinus roxburghii* (Sarja rasa or Chir pine), has been utilized for wound healing in traditional Ayurvedic medicine in India since ancient times. Ayurvedic literature characterizes Chir pine resin as possessing antimicrobial and cleansing properties, applied topically to disinfect wounds, promote healing, and eliminate maggots from infected sores. Healers applied warm turpentine oil, occasionally combined with ghee or camphor, to cuts and chronic ulcers to promote drying and tissue repair. Turpentine and pine resins have historically been esteemed in Ayurvedic balms, Greek poultices, and European surgical dressings for their infection-fighting properties and their role in facilitating tissue healing. This crosscultural continuity highlights the significant role of turpentine oil in premodern wound care as a natural antiseptic salve [47,48].

Another well-known natural product with regenerative potential is the turmeric plant (*Curcuma longa*), a herb in the ginger family that has historically served as a dietary spice and coloring agent in Indian and Chinese cuisines. The plant’s rhizome has been utilized for centuries in Indian and Chinese traditional medicine, serving as the most valuable component for medicinal applications. Curcumin is widely utilized in Indian traditional medicine for treating biliary disorders, cough, diabetic ulcers, hepatic disorders, rheumatism, and sinusitis [50]. Curcumin paste combined with lime is commonly used as a home remedy for inflammation and wound treatment. Curcumin’s wound healing potential is linked to its biochemical properties, including anti-inflammatory, anti-infectious, and antioxidant activities. Curcumin enhances cutaneous wound healing by participating in tissue remodeling, granulation tissue formation, and collagen deposition. Studies indicate that curcumin application on wounds enhances epithelial regeneration, fibroblast proliferation, and vascular density [51,52].

Table 3 outlines significant historical substances—spanning from ancient cultures to traditional remedies utilized to enhance skin healing. For each, we observe the geographic or cultural background, the asserted therapeutic effects (such as antimicrobial, anti-inflammatory, etc.), and relevant historical insights from classical references. A variety of these substances, such as honey and aloe, are recorded in ancient medical literature or archaeological findings, showcasing centuries of practical application.

Each of these substances exemplifies the use of botanicals and resins in traditional medical systems for wound treatment. Throughout various periods and locations, healers created poultices, infusions, and salves from natural substances to cleanse wounds, diminish inflammation, halt bleeding, and promote healing, establishing a significant historical basis for their ongoing application in wound care [60].

### 3.2. Key Natural Products

Natural products exhibit various biological activities, with a significant number of newly developed drugs originating from secondary metabolites and their derivatives.

**Figure 5:**
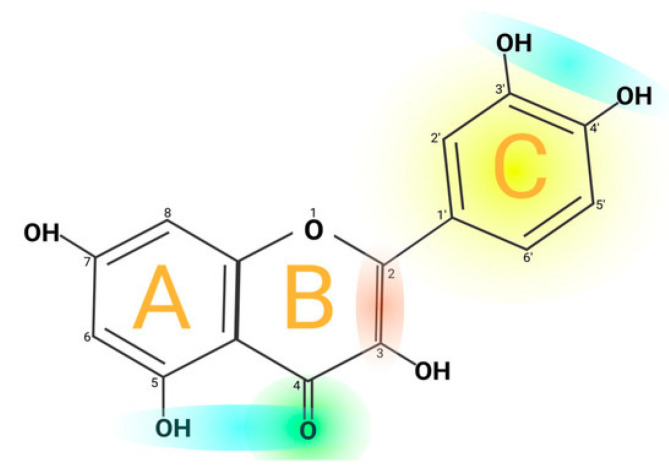
Chemical structure of quercetin. Reprinted from an open-access source [62].

Quercetin is a flavonoid (pentahydroxyflavone, C_15_H_10_O_7_) found in various plant parts, including the stems, leaves, flowers, skins, seeds, and fruits of apples, grapes, onions, tea, tomatoes, and *Ginkgo biloba* [61]. Quercetin primarily demonstrates anti-inflammatory and antioxidant properties while enhancing immune function. The structure presented in Figure 5 [62] highlights the three-ring backbone of quercetin. In particular, the catechol B-ring (adjacent OH groups at 3′–4′, colored yellow) and the C-ring 4-oxo/5-OH system (green/blue) are key for electron donation. These features (A-, B-, C-ring moieties) give quercetin its antioxidant properties. In vitro studies indicate that quercetin’s anti-inflammatory mechanism involves the inhibition of cyclooxygenase (COX) and lipoxygenase (LOX). Additionally, quercetin may promote inflammation by enhancing peroxisome proliferator-activated receptor C (PPAR-γ) activity, which indirectly mitigates inflammation and antagonizes NF-κB or the transcriptional activation of activator protein-1 inflammatory genes [61,63]. Research indicates that quercetin binding to collagen increases hydroxyproline concentration in granulation tissue, rising from 0.78 mg/mL in controls to 1.84 mg/mL, suggesting improved collagen production. The primary objective of quercetin nano dosage forms is to enhance permeation and stability due to quercetin’s poor water solubility. The present nanoformulation utilizing quercetin as the primary raw material has an age restriction [64].

**Figure 6:**
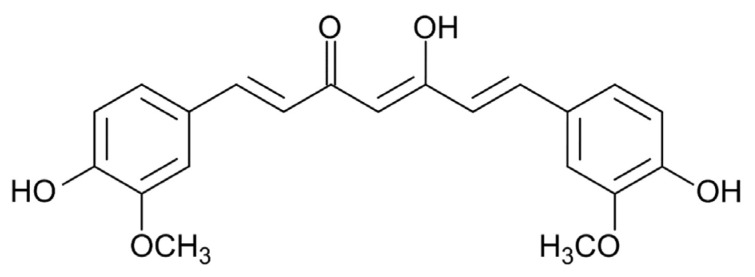
Chemical structure of curcumin. Reprinted from an open-access source [65].

Curcumin is a polyphenolic compound that can be found abundantly in the medicinal plant turmeric. Curcumin is a diferuloylmethane with a crystalline yellow-orange color, molecular weight of 368.39 g/mol, melting temperature of 183 °C, and with the chemical formula C_21_H_20_O_6_, as seen in Figure 6 [65].

Recent studies indicate that curcumin exhibits various pharmacological effects, such as anti-inflammatory, antioxidant, and antibacterial properties [66,67]. Curcumin may significantly contribute to wound healing through its antioxidant and anti-inflammatory properties. It may also influence four processes: granulation tissue formation, collagen deposition, tissue remodeling, and wound contraction, promoting wound healing. A study demonstrated that curcumin significantly upregulated the mRNA expression of collagen type I, keratinocyte growth factor-1, and epidermal growth factor receptor (EGFR) in an in vitro wound healing model using human gingival fibroblasts. The ERK signaling pathway is crucial for the expression of col1 and EGFR mRNA induced by curcumin. Gong et al. [68] conducted in vitro experiments to evaluate a curcumin thermosensitive gel dressing. Animal wound healing using curcumin heat-sensitive dressing demonstrated significant improvements, including enhanced re-epithelialization, organized granulation tissue, and increased fibroblast deposition [69]. Curcumin has been identified as a skin wound healing agent that promotes wound healing through the production of TGFβ1. GFβ1 stimulates fibroblasts in the surrounding tissue to proliferate and express integrin receptors (Level 3), facilitating their migration to the wound site and promoting healing (Level 2). The trend also recognized curcumin as a safe compound. Poor bioavailability owing to low water solubility and stability limits its clinical use. To improve wound healing and scar prevention, curcumin nanoplexes and chitosan–curcumin composites have been produced [70,71,72]. Clinical data studies indicated that topical curcumin demonstrated significant bioactivity, which may assist in the advancement of effective composite formulations [67,69].

*Centella asiatica*, or Asiatic pennywort, has a long history of use in promoting wound healing. It is found in Asia, particularly abundant in India, Pakistan, and Madagascar [73]. *Centella asiatica* enhances healing in skin conditions, including small wounds, scratches, burns, and hypertrophic wounds, while demonstrating anti-inflammatory and antibacterial properties. Extracts from the aerial parts of *Centella asiatica* have been shown to enhance the healing of chronic ulcers in Sprague Dawley rats regarding width, depth, and length. Rats with acute radiation dermatitis wounds exhibited earlier healing when treated with *Centella asiatica* extracts compared to the control group without treatment [74,75]. Triterpenes from *Centella asiatica* enhance collagen remodeling and glycosaminoglycan synthesis in a rat wound model. Oral administration of madecassoside from this natural product facilitated collagen synthesis and angiogenesis in a mouse wound model [76,77].

Asiaticoside is derived from the medicinal plant *Centella asiatica*. Kreimendahl et al. [78] propose that asiaticoside enhances wound healing by promoting fibroblast proliferation, potentially linked to tissue cell migration around the wound or the expression and activation of specific growth factors (Level 3). Asiaticoside promotes col1 synthesis in human dermal fibroblasts via Smad 2 and Smad 3 phosphorylation, leading to Smad 3 and Smad 4 binding. Both in vivo and in vitro studies demonstrated that asiaticoside has significant healing properties in normal and chronic wound models (Level 2) [73].

Oleanolic acid, a pentacyclic triterpene sourced from *Olea europaea* and *Ligustrum lucidum*, inhibits fibroblast proliferation and collagen synthesis through the suppression of P311 gene expression and a reduction in TGF-β1 signaling. It triggers fibroblast apoptosis through mitochondrial membrane disruption and caspase activation, thereby inhibiting excessive scar tissue formation. Research indicates that it facilitates ECM remodeling through upregulating MMP-2 activity and downregulating TIMP-1 levels, thereby promoting scar-free healing [79].

Vitamin C facilitates wound healing by modulating the immune response and modifying collagen, reducing excessive proinflammatory signals while enhancing fibroblast proliferation and substantial extracellular matrix accumulation (Level 3) [80,81]. Vitamin E extends its role beyond antioxidant protection by modifying gene expression, such as upregulating connective tissue growth factor, which facilitates fibroblast-mediated healing and diminishes inflammation (Level 3), thereby positively affecting scar formation during remodeling (Level 2) [82].

The bioactive compounds of *Aloe vera* (e.g., aloesin) function at various stages: they promote initial angiogenesis and an anti-inflammatory response (increasing TGF-β and other cytokines), expedite granulation tissue formation and re-epithelialization, and elevate growth factor levels (FGF, VEGF, KGF-1) that enhance cell migration and matrix synthesis (Levels 3 and 2) [83,84].

Hibiscus extracts have shown improved transition from inflammation to proliferation by enhancing macrophage activity in wounds, promoting angiogenesis, and augmenting collagen fiber deposition via growth factor pathways such as VEGF and TGF-β1 [85], addressing Level 3 of the conceptual healing hierarchy framework. The constituents of thyme essential oil provide antibacterial protection, inhibit proinflammatory cytokines, and promote fibroblast migration, proliferation, and collagen synthesis in the wound bed, thereby facilitating expedited tissue regeneration during the proliferative phase. Pomegranate polyphenols have anti-inflammatory properties that facilitate the rapid closure of wounds and enhance the proliferative phase; pomegranate extracts have been shown to elevate fibroblast density, neovascularization, and keratinocyte proliferation, hence accelerating re-epithelialization [83,85].

The antibacterial effects of honey are believed to stem from its capacity to produce hydrogen peroxide, a recognized antimicrobial agent. Upon contact with wound exudate, honey becomes diluted, therefore activating the enzyme glucose oxidase, which generates low, non-toxic concentrations of hydrogen peroxide. Honey dressings serve several functions, including: enhancing patient comfort and mobility, controlling moderate to severe exudate and excessive fluid, safeguarding granulation tissue and addressing infections—specifically *P. aeruginosa*, *S. aureus*, *C. albicans*, *E. coli*, and strains resistant to methicillin and vancomycin [86].

Propolis, a resin produced by bees, exhibits extensive efficacy throughout all stages of healing: it may facilitate hemostasis (potentially by promoting initial clot formation), reduce oxidative stress and excessive inflammation through its flavonoids, and expedite proliferation and remodeling by activating fibroblasts, fostering type I/III collagen deposition and reorganization, and aiding re-epithelialization. These are only chosen instances of bioactive substances, each distinctly enhancing one or more stages of the wound healing cascade via immunomodulatory, growth-factor-activating, and tissue-regenerative mechanisms [83].

Other natural products showing promise in soft tissue healing include, but are not limited to, *Ficus septica* Burm.f. latex [87], thyme oil [88], sesame oil [89], milk thistle extract (silymarin) [90], *Calendula officinalis* extract [91], royal jelly [92], bee pollen [93], and microalgae [94]. Table 4 presents compounds that have been identified to modulate inflammation, oxidative stress, or growth factor signaling in skin repair (Level 3).

### 3.3. Categories of Compounds of Interest for Tissue Healing

#### 3.3.1. Antioxidants

Antioxidants are naturally occurring polyhydroxylated phenolic compounds distinguished by their low molecular weights. Specific cellular enzymes are situated in compartments with considerable antioxidant properties that neutralize radicals. Many plants and fruits include dietary polyphenols with antioxidant characteristics, including flavonoids, phenolic acids, tannins, lignans, stilbenes, catechins, and carotenoids. Antioxidants inhibit the intracellular oxidation of substances. This process (Level 3) involves the extraction of electrons or hydrogens from a material, therefore mitigating oxidative damage to a cell by direct contact with radicals. The position and number of hydroxyl groups on the aromatic rings of these antioxidants may substantially affect their antioxidant efficacy. Antioxidants act as radical scavengers, mitigating oxidative damage caused by reactive oxygen species (ROS). Antioxidants may neutralize ROS from internal or external sources [99]. Excessive ROS induce oxidative stress, which fosters proinflammatory conditions. Antioxidants, such as polyphenols, are chemicals that may donate electrons to ROS, thereby averting the depletion of electrons from physiologically important molecules like proteins or DNA. Antioxidants catalyze processes that transform ROS into stable molecules, preserving non-toxic ROS levels in damaged tissues and promoting healing [100,101].

For better clarity, only a selection of antioxidants will be referenced. Astaxanthin, a marine-derived carotenoid, serves as a potent antioxidant that aids in reducing oxidative damage in wounds. In a burn wound model, astaxanthin reduced ROS-induced tissue damage by downregulating ROS-producing enzymes such as xanthine oxidase and NADPH oxidase [102]. This decrease in oxidative stress results in improved healing outcomes: wounds in mice treated with astaxanthin exhibited accelerated closure (Level 2), accompanied by significantly enhanced collagen I deposition (Level 3) and elevated levels of growth factors (Level 3), including basic fibroblast growth factor (bFGF), in the regenerating tissue. Astaxanthin may enhance healing quality (Level 1); postoperative studies indicate that its administration can diminish fibrosis and scar formation (Level 2), as shown by reduced subepithelial collagen deposition in healing mucosal wounds (Level 3). Astaxanthin enhances re-epithelialization by regulating excess ROS and inflammation, resulting in stronger and less fibrotic wounds [103].

Silibinin, the active flavonolignan in milk thistle extract (silymarin), demonstrates significant antioxidant properties that aid wound healing. Studies on burn injury models indicate that silymarin administration, whether topical or systemic, can mitigate oxidative stress resulting from burns. It significantly reduces malondialdehyde (MDA), a marker of lipid peroxidation and inflammatory mediators, while restoring endogenous antioxidants such as glutathione in the affected burn tissue [90]. Treated animals exhibited decreased neutrophil infiltration (reduced myeloperoxidase activity) and enhanced tissue morphology, suggesting diminished oxidative damage and inflammation. Silibinin facilitated improved healing of burn wounds (Level 2), indicating its potential as an adjunct therapy to reduce oxidative injury and enhance tissue regeneration in burns. Silibinin may mitigate oxidative damage, thereby potentially reducing inflammation and scarring in wound healing [90], yet further in-depth clinical studies are needed to better comprehend patient outcomes (Level 1).

**Figure 7:**
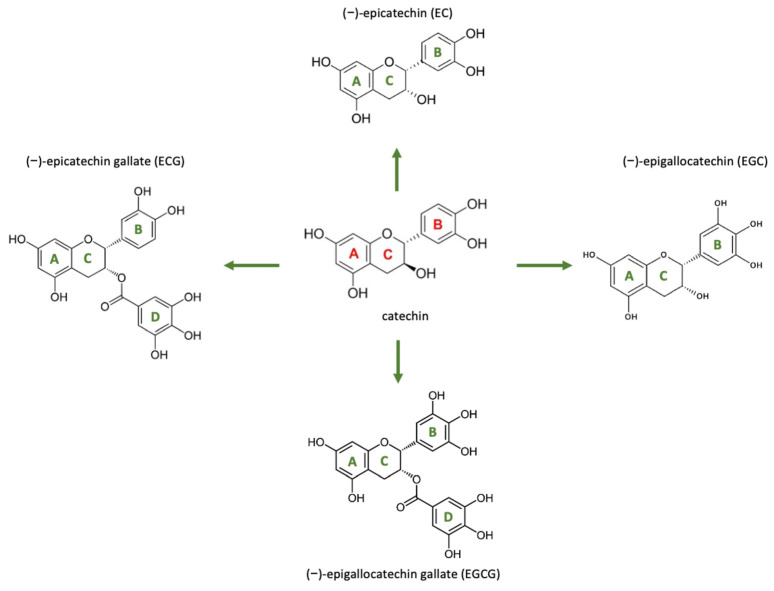
The chemical structures of the four primary catechins present in green tea along with their precursor. Reprinted from an open-access source [104].

EGCG, a catechin monomer present in green tea (*Camellia sinensis*), is recognized for its antioxidant and antifibrotic properties (Level 3). EGCG features a structure composed of three aromatic rings (A, B, and D) linked by a pyran ring (C), as illustrated in Figure 7 [104]. In its unaltered state, it manifests as a white or light pink powder or crystalline material devoid of any scent. EGCG dissolves in various solvents including water, ethanol, methanol, acetone, tetrahydrofuran, and pyridine, and has a melting point of 218 °C. The distinctive and readily adjustable chemical composition is mainly accountable for its biological functions and health advantages [104].

It modulates fibrosis-related pathways through the inhibition of VEGF, connective tissue growth factor (CTGF), and TGF-β1 (Level 3), which are essential mediators of pathological fibrosis. EGCG influences wound healing during the inflammatory and proliferative phases by modulating macrophage activity, decreasing mast cell activation, and promoting the M2 macrophage phenotype, which is linked to tissue repair rather than fibrosis. Research indicates that EGCG inhibits the synthesis of collagen type I (COL-I) by disrupting the PI-3K/Akt/mTOR signaling pathway, which is crucial for fibroblast activation and ECM deposition. Furthermore, EGCG modifies the gene expression of mesenchymal stem cells (MSCs), thereby improving their antifibrotic characteristics, specifically through the reduction of TGF-β1 and the elevation of TGF-β3 expression. EGCG has demonstrated potential in clinical settings for reducing skin thickness, enhancing elasticity, and preventing the formation of hypertrophic scars (Level 2). Nonetheless, its limited bioavailability resulting from instability in alkaline environments requires localized application to achieve optimal therapeutic results [105].

Gallic acid (GA), a naturally occurring polyphenol present in *Rhus coriaria*, witch hazel, and oak bark, demonstrates significant antioxidant, anti-inflammatory, and antifibrotic properties. It inhibits fibroblast contraction and α-smooth muscle actin (α-SMA) expression via the RhoA/ROCK signaling pathway (Level 3), thereby preventing myofibroblast differentiation, which is a crucial event in scar formation. Gallic acid induces apoptosis in hypertrophic scar fibroblasts (HSFs) through the Bcl2/Bax mitochondrial-dependent pathway and causes necrosis via calcium elevation and lysosomal membrane rupture. Furthermore, it inhibits the Toll-like receptor 4 (TLR-4)/NF-κB pathway, leading to a decrease in proinflammatory cytokines, including TNF-α, IL-6, IL-1β, and IL-8. Gallic acid contributes to scar reduction and improved tissue remodeling by downregulating profibrotic pathways [15,106].

These natural antioxidants enhance wound healing outcomes by mitigating oxidative stress at the wound site. Their effects include diminished scarring (attributable to decreased oxidative damage to matrix proteins and regulated fibroblast activation), augmented collagen production (by maintaining fibroblast functionality and stimulating prohealing signaling pathways), and expedited re-epithelialization (Levels 3 and 2).

#### 3.3.2. Anti-Inflammatory

The inflammatory phase is crucial in wound healing; however, excessive and prolonged inflammation in the early stages can lead to scarring, fibrosis, and delayed healing. Furthermore, inflammation is crucial in preventing microbial infections and eliminating dead cells and cellular debris. At this stage, proinflammatory mediators, including interleukins, TNF-α, inducible nitric oxide synthase, and chemokines, are produced. Proinflammatory M1 phenotype macrophages are the initial responders in the repair stages. The prorepair, anti-inflammatory M2 phenotype is more prevalent in the later stages of wound healing [107].

Natural compounds, especially those derived from plants, have garnered significant attention for their ability to modulate the inflammatory response during wound healing. These chemicals often have anti-inflammatory and antioxidant characteristics, allowing them to address several facets of the wound environment. Curcumin, a polyphenolic substance obtained from *Curcuma longa* (turmeric), has been well-researched for its potent anti-inflammatory properties. It regulates the activity of nuclear factor-kappa B (NF-κB), a transcription factor that governs the expression of several proinflammatory genes, thereby diminishing cytokine production and oxidative stress [108]. Preclinical studies indicate that curcumin accelerates wound healing, enhances collagen production, and improves tissue regeneration. Likewise, the polyphenols found in green tea, particularly EGCG, have shown efficacy in inhibiting inflammatory mediators and functioning as antioxidants. EGCG suppresses the activation of inflammatory pathways and reduces the synthesis of cytokines such as IL-6 and TNF-α, fostering a more conducive environment for tissue healing [109].

Propolis, a resinous substance gathered by bees, is abundant in flavonoids and phenolic acids and has shown considerable anti-inflammatory properties. Research indicates that propolis extracts may diminish inflammatory cell infiltration, facilitate re-epithelialization, and augment granulation tissue development. Another significant example is *Centella asiatica*, a traditional medicinal herb extensively used for wound treatment. The active ingredient, madecassoside, has been shown to diminish inflammation and promote fibroblast proliferation and collagen synthesis. The effects are mediated via the modulation of TGF-β signaling and the downregulation of proinflammatory cytokines [110]. The combined anti-inflammatory and regenerative properties of *C. asiatica* make it a viable option for the treatment of both acute and chronic wounds. Triterpenoids such as friedelin, extracted from diverse plant sources, have antibacterial and anti-inflammatory effects that are especially advantageous for treating infected or slow-healing wounds. Friedelin has been shown to inhibit proinflammatory cytokines and promote wound contraction, particularly in wounds infected with methicillin-resistant *Staphylococcus aureus* (MRSA), providing a natural alternative to traditional antimicrobials. Integrating these natural substances into wound care formulations—such as gels, ointments, and hydrogel dressings—has shown encouraging outcomes in both preclinical and clinical environments [111,112]. A topical preparation combining curcumin and honey markedly enhanced burn wound healing by reducing inflammation and facilitating tissue regeneration. Additionally, biopolymer-based dressings laced with natural anti-inflammatory drugs have been created to improve wound covering and healing efficacy, providing prolonged release and targeted action [112].

#### 3.3.3. Antimicrobials

Skin wound infections are categorized into minor superficial infections and severe, life-threatening infections, depending on their cause and the extent of microbial invasion. Initially, Gram-positive organisms such as *Staphylococcus aureus* and *Streptococcus pyogenes* predominate in the wound. In advanced stages, Gram-negative organisms like *Escherichia coli* and *Pseudomonas aeruginosa* are associated with developing chronic wounds. The immune system’s rapid response is essential for preventing infection during wound repair by removing invading pathogens [113]. Macrophages move to the wound site to phagocytize bacteria, while helper T cells release interferon-γ and CD40 ligands, orchestrating the adaptive and humoral immune responses to eradicate pathogens. Failure of the immune system to eliminate pathogens results in infection, which degrades granulation tissue, growth factors, and extracellular matrix components, disrupting normal healing [114,115].

Infected wounds present a considerable challenge owing to ongoing microbial invasion and uncontrolled inflammation. Wound infections are estimated to represent 60–80% of all bacterial infections in humans. The presence of antibiotic-resistant pathogens and protective biofilms in chronic wounds complicates treatment, as bacteria within biofilms resist immune responses and standard antibiotics. There is increasing interest in natural compounds that enhance host immunity and provide direct antimicrobial effects to facilitate the healing of infected wounds. This discussion focuses on a few compounds, such as berberine, allicin, tea tree oil, andrographolide, and baicalin, highlighting their mechanisms of action and significance in wound infection therapy [116].

Berberine is an isoquinoline alkaloid derived from plants such as Berberis and Coptis species, known for its extensive pharmacological properties [117]. Numerous studies indicate that berberine exhibits significant antimicrobial properties and anti-inflammatory effects. Berberine disrupts pathogens by inhibiting bacterial cell division, damaging structural integrity, and interfering with microbial metabolism. These actions render it effective against challenging wound pathogens; for instance, berberine can notably inhibit *Staphylococcus aureus*’s growth and biofilm formation, including antibiotic-resistant strains. Berberine’s immunomodulatory effects are also significant. It reduces excessive inflammation by inhibiting proinflammatory cytokines (IL-1, IL-6, TNF-α) by suppressing the MAPK/NF-κB signaling pathways and decreasing COX activity to lower prostaglandin production. In an infected wound, this results in a quicker resolution of the inflammatory phase and a beneficial change in macrophage responses; berberine has been demonstrated to promote the transition of macrophages from a proinflammatory M1 phenotype to a prohealing M2 phenotype, thus enhancing the local wound environment. Berberine’s combined antimicrobial and anti-inflammatory properties (Level 3) have shown efficacy in accelerating the healing of infected wounds by reducing bacterial load, controlling excessive inflammation, and enhancing tissue regeneration, including re-epithelialization and angiogenesis, in wound models (Level 2). These findings highlight berberine’s potential as a therapeutic adjunct in managing infected wounds [116,117], encouraging its evaluation in clinical settings for determining how its utilization can improve the function and appearance of real patients’ wounds (Level 1).

Allicin, a sulfur-containing compound derived from garlic (*Allium sativum*), is recognized for its antimicrobial properties and its effects on the immune system. It exhibits extensive antibacterial activity against both Gram-positive and Gram-negative bacteria, including antibiotic-resistant strains such as methicillin-resistant *S. aureus* (MRSA) [118]. Allicin exerts its bactericidal effect by reacting with thiol groups in microbial proteins and enzymes, disrupting essential metabolic processes in pathogens. Allicin inhibits crucial bacterial respiration and metabolism by oxidatively modifying cysteine residues in enzymes and inactivating cofactors such as coenzyme A, resulting in growth arrest or cell death. The thiol-targeting mechanism supports garlic’s traditional role as an antiseptic and accounts for allicin’s efficacy in lowering wound infection rates noted in animal studies. Besides its direct antimicrobial properties, allicin demonstrates notable immunomodulatory and anti-inflammatory effects that enhance wound healing. This can modulate the acute inflammatory response by preventing neutrophil migration into tissues and reducing the release of proinflammatory cytokines associated with TNF-α signaling [118]. These measures mitigate tissue damage and chronic inflammation in wounds. Simultaneously, allicin may bolster specific immune functions; it has been noted to activate signaling pathways (e.g., p21^ras^-ERK) in lymphocytes and macrophages, potentially enhancing their ability to clear pathogens. The overall result is a regulated inflammatory environment that allows for effective infection defense. Allicin notably seems to enhance the reparative phase of wound healing. Topical garlic preparations in animal models markedly enhanced fibroblast proliferation in wound tissue (Level 3), resulting in more organized and expedited tissue repair (Level 2). This healing activity and allicin’s ability to eliminate wound pathogens and decrease infection accounts for the observed acceleration of wound closure and reduced infection rates with garlic extracts in vivo. Therefore, garlic rich in allicin is a versatile agent in managing infected wounds, exhibiting antimicrobial, anti-inflammatory, and proregenerative properties [118].

Tea tree oil (TTO), derived from *Melaleuca alternifolia*, has historically been utilized as a topical antiseptic and anti-inflammatory agent. The antimicrobial spectrum of TTO is broad, demonstrating significant efficacy against various bacteria, including *S. aureus*, *E. coli*, *P. aeruginosa*, and MRSA, fungi, and certain viruses. Tea tree oil primarily exerts its antibacterial effects by disrupting microbial membrane integrity. The terpene components of TTO exhibit high lipophilicity, integrating into bacterial cell membranes and resulting in compromised membrane function and structure [119]. Research indicates that TTO exposure results in ion leakage (e.g., K^+^) and the release of cellular contents from bacteria, inhibits respiratory functions, enhances cell permeability to dyes, and causes morphological damage to cells. TTO induces considerable leakage of 260-nm-absorbing materials in S. aureus, suggesting the release of nucleic acids or nucleotides, and increases the bacteria’s vulnerability to osmotic stress, thereby confirming a mechanism of action related to membrane disruption [119,120]. The swift bactericidal properties of tea tree oil render it effective in managing wound pathogens, with clinical studies investigating TTO formulations for MRSA decolonization in infected patients. Tea tree oil possesses direct antimicrobial properties and significant anti-inflammatory and immunomodulatory effects that facilitate wound healing. TTO, especially its active component terpinene-4-ol, can inhibit excessive inflammation in skin injuries by regulating the vascular and cellular phases of the inflammatory response. The topical application of TTO reduces histamine-induced edema and mitigates wheal-and-flare skin reactions, suggesting its capacity to limit vasodilation and plasma extravasation in inflammatory responses. In mouse models, TTO reduced swelling in allergic contact dermatitis and diminished ROS production by activated immune cells while preserving normal immune function. This balanced anti-inflammatory effect can avert tissue damage from prolonged inflammation while allowing essential immune responses to occur (Level 3). Clinical and in vivo studies indicate that tea tree oil may enhance wound healing by managing infection and inflammation. TTO treatment in wound care studies has been linked to quicker healing times and better outcomes (Levels 2 and 1), reinforcing its function as a multifunctional adjunct in infected wound therapy. Tea tree oil functions as an antimicrobial agent that diminishes wound bioburden and as an anti-inflammatory agent that fosters an environment favorable for tissue repair [119,120].

Andrographolide is a diterpenoid lactone derived from *Andrographis paniculata*, commonly referred to as the “king of bitters,” and is traditionally recognized for its anti-infective and anti-inflammatory properties. Recent studies confirm that andrographolide exhibits immunostimulatory effects and notable antibacterial and antiviral activities. The diverse mechanisms of action are especially pertinent to persistent wound infections associated with biofilms and resistant pathogens. Andrographolide primarily influences bacterial virulence and the host–pathogen interaction instead of functioning as a typical bactericidal agent [121]. For instance, andrographolide disrupts the quorum-sensing mechanisms in *P. aeruginosa*, a prevalent wound pathogen, which governs biofilm development and the production of virulence factors. It reduces the expression of polysaccharide synthesis genes (such as Psl) to inhibit biofilm matrix formation and prevents the production of virulence factors like pyocyanin while also suppressing the MexAB-OprM efflux pump to decrease the bacterium’s drug resistance. In *S. aureus* infections, andrographolide primarily exerts its protective effect by modulating the host immune response rather than through direct bactericidal action. It effectively reduces excessive inflammation by inhibiting the release of IL-6, TNF-α, and other cytokines, partly by blocking NF-κB activation in immune cells. This results in diminished tissue damage and aids in containing the infection. Andrographolide concurrently influences *S. aureus* by downregulating the bacterial regulator SarA, thereby reducing biofilm formation and the expression of several staphylococcal virulence genes [121]. Andrographolide has been demonstrated to inhibit communication signals such as autoinducer-2 (quorum sensing) in *E. coli* and other bacteria, diminish bacterial adhesion to host cells, and directly damage bacterial cellular structures, including effects on cytoskeletal elements. These actions diminish the microbes’ strength, increasing their vulnerability to elimination. Andrographolide notably stimulates the host’s antimicrobial defenses by enhancing the phagocytic activity of macrophages and other immune cells, thus accelerating bacterial elimination in vivo. A study demonstrated that an andrographolide formulation with enhanced bioavailability exhibited a limited direct bacteriostatic effect in vitro, yet significantly increased survival in an animal infection model, which was attributed to immune stimulation by andrographolide. The dual function of neutralizing pathogens and enhancing host immunity is crucial in wound healing, as controlling infection and resolving inflammation are essential for tissue repair. Andrographolide reduces bacterial virulence and inflammatory damage, facilitating more effective wound healing. The traditional application in infection-related conditions is supported by a solid scientific basis for its use in managing infected wounds [121]. Thus, andrographolide has a great potential for solving the pressing problem of infected wounds, with encouraging results for moving toward clinical testing (Level 1).

Baicalin, a flavone glycoside from the roots of *Scutellaria baicalensis* (Chinese skullcap), is noteworthy for infected wounds due to its antimicrobial and immunomodulatory properties. Baicalin exhibits notable anti-inflammatory and antibacterial effects, along with antioxidant properties. In herbal medicine, the therapeutic efficacy of *Scutellaria* is primarily linked to its ability to modulate the host immune response, enhancing immune defense and reducing harmful inflammation [122]. Baicalin seems to influence both the pathogen and the immune response in wound infections. Baicalin directly inhibits microorganisms and their biofilms. Research indicates that baicalin exhibits antibacterial properties against *S. aureus* and other bacteria, demonstrating the ability to inhibit biofilm formation and eliminate established biofilms at adequate concentrations. Baicalin has been shown to disrupt the biofilm of Acinetobacter, a drug-resistant genus, and likely impacts staphylococcal biofilms by interfering with extracellular polysaccharides or quorum-sensing systems. Antibiofilm activity is essential for pathogens in chronic wounds. Conversely, baicalin significantly reduces inflammatory responses. It prevents the release of proinflammatory mediators from immune cells; specifically, baicalin (and its aglycone baicalein) can stabilize mast cells and inhibit the secretion of histamine, IL-1β, IL-6, and other inflammatory signals that could worsen tissue damage [122,123]. Baicalin mitigates edema, pain, and collateral damage in the wound area by inhibiting the NF-κB pathway and associated inflammatory cascades. Baicalin may enhance the host’s inherent antibacterial defenses. Recent studies indicate that baicalin can influence mitochondrial function in host cells, thereby improving bacterial clearance and augmenting the bactericidal activity of immune cells during infections. In vivo studies indicate that baicalin treatment safeguards animals against MRSA infection, which is associated with decreased inflammatory cytokines and enhanced bacterial clearance, suggesting an immune-regulating mechanism alongside direct microbicidal effects (Level 3). Baicalin’s capacity to eliminate or inhibit pathogens while moderating excessive inflammation underscores its significance in wound healing. It fosters a balanced immune response that eliminates the infection while minimizing tissue damage. Wound dressing studies utilizing baicalin or baicalein have demonstrated enhanced healing, attributed to the compounds’ antimicrobial, anti-inflammatory, and antioxidant properties that support tissue recovery (Level 2). Baicalin serves as a natural product that simultaneously interacts with the immune system and microbes, guiding infected wounds toward resolution [122,123,124]. However, it remains unclear whether this success leads to fewer wound care revisions or better patient-reported aesthetic satisfaction (Level 1), imposing the need for in-depth patient-based studies to clarify these aspects.

## 4. Natural Materials for ECM Restoration

Biomaterials that consist of isolated ECM components represent the most fundamental form of ECM-derived biomaterials utilized in tissue repair. ECM-derived biopolymers, including collagen, hyaluronic acid (HyA), fibrin, and gelatin, are commonly crosslinked through chemical or physical methods to create a structure that promotes cell infiltration and tissue repair. These biopolymers are well-suited for wound repair due to their occurrence in the native extracellular matrix, biocompatibility, biodegradability, and low immunogenicity [125]. Most studies involving natural polysaccharides, structural proteins, and peptides demonstrate effects on healing dynamics (e.g., angiogenesis, ECM deposition, granulation) and molecular pathways (Level 2 and Level 3, respectively) but often lack direct evidence of clinical-level improvements (Level 1), such as functional restoration or scar reduction that meets application-specific thresholds.

### 4.1. Polysaccharides

Hyaluronic acid (HyA) is a naturally occurring biopolymer derived from the extracellular matrix, recognized for its capacity to enhance wound healing and modulate inflammation, angiogenesis, and cell migration. Additionally, HyA has been extensively utilized in developing biomaterials, particularly hydrogels, which exhibit significant potential for tissue repair [126,127,128]. The hydrophilic properties of HyA are essential in the formulation of wound healing therapies, as they help sustain a moist wound environment, thereby promoting epithelial migration. Various forms of HyA-derived biomaterials, such as electrospun scaffolds and hydrogels, have been evaluated for their wound healing efficacy, similar to collagens [125]. Dermal fibroblast infiltration occurs within HyA-based scaffolds in a manner dependent on porosity, indicating its potential as a template for tissue integration and wound healing. The implantation of electrospun HyA scaffolds in full-thickness mouse wounds enhanced vascularization and re-epithelialization compared to silicon dressings. Hyaff-11, a HyA-based wound dressing, has demonstrated effective ECM deposition and endothelial cell proliferation in vitro, achieving a reduction of up to 50% in DFU wound size, alongside increased endothelial cell proliferation and ECM deposition (collagens, fibronectin, and laminins) in vivo [100,126].

Chitosan is a positively charged polyelectrolyte and the only basic amino polysaccharide found in nature. This linear copolymer comprises d-glucosamine and N-acetyl-d-glucosamine, connected by β-1,4-glycosidic bonds, with a molecular weight ranging from 100 to over 1000 kDa [50,129]. Chitosan exhibits several inherent advantages, including biodegradability, biocompatibility, bioactivity, and low immunogenicity. The antibacterial properties of chitosan are attributed to its positive charge, which enhances its interaction with negatively charged components in bacterial membranes, including anionic polysaccharides, proteins, and nucleic acids [130]. Chitosan is insoluble in neutral and alkaline aqueous solutions with pH values exceeding 6.5, significantly limiting its applicability. This copolymer has been incorporated into various formulations, including nanoparticles, hydrogels, micelles, and hyaluronic/oleic-acid-loaded systems, as well as through the glucosylation of hydrophobic molecules in preclinical studies to enhance its bioavailability [129,130,131].

Natural polysaccharide biomaterials, including alginate, dextran, and cellulose, are integral to the restoration of the ECM. They significantly influence fibroblast activity and facilitate matrix remodeling throughout the wound healing process. Alginate-based dressings establish a moist, provisional matrix that facilitates the migration and proliferation of fibroblasts, ultimately forming robust granulation tissue and the deposition of collagen. Furthermore, calcium alginate gels possess the capability to sequester excessive proteases present in wound exudate, including matrix metalloproteinases [132]. This action safeguards newly synthesized collagen from premature degradation [133]. Dextran-derived scaffolds play a significant role in facilitating the proliferative phase of healing by enhancing the organization of the collagen matrix and promoting the synthesis of type III collagen in fibroblasts. During the remodeling phase, specific sulfated dextran compounds play a crucial role in modulating extracellular matrix turnover by enhancing the activity of matrix metalloproteinase-2 (MMP-2), which facilitates regulated matrix remodeling [132]. These compounds can induce apoptosis in myofibroblasts in a timely manner, thereby preventing excessive scar formation [134]. Cellulose-derived biomaterials, such as bacterial nanocellulose and oxidized regenerated cellulose, serve as a structural reinforcement, functioning as a biocompatible fibrous scaffold that promotes the infiltration of cells [134]. The intricate porous nanofiber architecture of cellulose dressings facilitates extensive fibroblast ingrowth and promotes the organized deposition of collagen fibers across the entirety of the wound bed. Concurrently, oxidized cellulose demonstrates a positive biochemical effect: it effectively binds and neutralizes harmful proteases and free radicals while maintaining the presence of growth factors within the wound environment. The dual function of this approach plays a crucial role in sustaining an advantageous microenvironment conducive to extracellular matrix (ECM) assembly [2]. This, in turn, leads to expedited granulation and enhanced quality of tissue remodeling. For instance, the application of oxidized regenerated cellulose/collagen dressings has been shown to markedly enhance fibroblast proliferation and promote wound closure in vivo within models characterized by impaired healing processes. Alginate, dextran, and cellulose work in concert to facilitate the process of wound healing. They function as bioactive extracellular matrix analogs, providing structural scaffolding for forming new tissue [134]. Additionally, these substances play a crucial role in regulating collagen synthesis and matrix turnover (Level 3), achieved through the stimulation of fibroblasts and the inhibition of proteases, as seen in Table 5. Moreover, evidence is also increasingly built up for Levels 2 and 1, with polysaccharides displaying promising in vivo and clinical outcomes.

### 4.2. Proteins

Collagen type I is the predominant element of the extracellular matrix and is extensively utilized as a biomaterial owing to its recognized ability to promote cell migration, infiltration, and proliferation. Collagen-based biomaterials have been explored in various forms for treating diabetic foot ulcers, including scaffolds, gels, particles, and films. Collagen type I electrospun matrices exhibited improved keratinocyte adhesion, suggesting their potential role in facilitating wound healing. Additionally, collagen derived from fish skin was electrospun to create a nanofibrous mesh, which promoted human keratinocyte adhesion, proliferation, and differentiation in vitro [139]. This study further demonstrated electrospun collagen’s biocompatibility and proregenerative capacity in wound re-epithelialization, as evidenced by findings in a murine model. Collagen-based biomaterials facilitate cell delivery, evidenced by MSCs seeded in collagen type I scaffolds, which improved healing in rabbit diabetic foot ulcer (DFU) models via enhanced angiogenesis and accelerated wound closure. Additional clinical studies demonstrating the effectiveness of collagen-based biomaterials in wound healing include the Promogran^®^ sponge (3M, St. Paul, MN, US), composed of collagen and oxidized regenerated cellulose. The combination of oxidized regenerated cellulose and collagen has demonstrated effectiveness in enhancing hemostasis and addressing intraoperative bleeding, which supports its role in wound healing [140,141].

Gelatin, also known as hydrolyzed collagen, is a product of the extracellular matrix that has been utilized as a biomaterial for wound healing. Due to its bioactivity, biodegradability, and modifiable characteristics, it is especially appropriate for wound healing applications. Gelatin hydrogels serve as effective cell delivery vehicles for wound healing, demonstrating laminin and fibronectin deposition in vitro alongside enhanced wound closure, re-epithelialization, and vascularization in preclinical mouse models [142].

Silk fibroin (SF) derived from Bombyx mori silkworms is a widely available natural fiber that can be obtained efficiently and cost-effectively. SF exhibits enhanced biocompatibility and biodegradability as a biomaterial, exhibiting great potential in applications of tissue engineering and drug delivery systems [143,144]. The convenient regeneration, excellent biocompatibility, notable mechanical properties, and versatile biodegradability of SF have been studied for the preparation of various products, including films, spongy matrices, and hydrogels, and have been evaluated for applications in tissue engineering. SF nanoparticles have been effectively engineered to regulate the release rate of biomolecules in a continuous manner with high stability. This review acknowledges advancements in SF-based drug delivery, in vitro engineering, and rejuvenation, highlighting the potential for further progress in these domains [144].

Keratin and fibrin are protein-based materials that serve essential functions in wound healing, acting both as natural healing agents and designed wound coverings. Fibrin, generated from fibrinogen during the clotting process, establishes the initial provisional matrix that facilitates hemostasis and acts as a scaffold for cell adhesion and migration. The fibrin clot serves a dual purpose: it stops bleeding while also capturing platelets, leukocytes, and growth factors, which in turn influences inflammation and aids in tissue repair [145]. A structural network is established that directs fibroblasts and endothelial cells into the wound; these fibroblasts ultimately break down the fibrin matrix and substitute it with a collagen-rich extracellular matrix during the remodeling phase. The initial role of fibrin as a physical barrier is crucial, as it aids in trapping microbes and offers protection against infection. In clinical applications, fibrin is employed in sealants and serves as a scaffold in skin grafts or tissue-engineered constructs to harness its inherent healing capabilities [146]. Keratin, a structural protein prevalent in skin (such as hair and wool), has gained recognition as an effective wound healing material owing to its bioactive properties and compatibility with biological systems [147]. Biomaterials derived from keratin, whether from human hair or animal wool, possess integrin-binding motifs like RGD sequences that enhance cellular attachment and proliferation, promoting cell adhesion and fibroblast infiltration. When applied to a wound, keratin has the potential to enhance hemostasis by reducing the time it takes for clotting to occur. Additionally, keratin dressings influence the inflammatory phase by directing macrophages to adopt a prohealing M2 phenotype, characterized by increased levels of anti-inflammatory IL-10 and reduced proinflammatory cytokines. During the proliferative phase, keratin-based materials enhance the processes of re-epithelialization and angiogenesis by increasing the expression of wound-associated keratin proteins and growth factor signaling that promote the formation of new tissue. This bioactive support promotes strong granulation tissue while minimizing inflammation—for example, keratin-based hydrogels have demonstrated enhanced collagen deposition and a decrease in scar-forming TGF-β signaling [147]. Keratin-based wound dressings have been created in various formats, such as hydrogels, films, and electrospun nanofibers, showing enhanced wound closure rates and excellent biocompatibility in preclinical research. In conclusion, fibrin primarily facilitates initial wound stabilization and serves as a temporary scaffold for repair, whereas keratin-based substances enhance healing throughout all stages (inflammation, proliferation, and remodeling) by effectively encouraging cell recruitment, matrix remodeling, and tissue regeneration. Each utilizes a natural structural function—fibrin serving as the temporary extracellular matrix of the clot and keratin providing the structural foundation of skin—to improve wound-healing results in contemporary wound management [148].

Overall, protein-based scaffolds support cellular attachment and tissue remodeling. While their biological activity has been extensively demonstrated in vitro and in animal models (Level 3), the extent to which they influence clinically meaningful healing outcomes remains uncertain and may vary by wound type.

### 4.3. Bioactive Peptides

Bioactive peptides and proteins (BAPPs) are functional molecules characterized by distinct amino acid sequences, playing crucial roles in various biological processes such as biocatalysis, immunomodulation, modulation of signaling pathways, and regulation of cellular fate and behavior [149]. Consequently, they exhibit significant promise as therapeutic agents for various conditions, including diabetes, tissue injury, cancer, infections, and chronic pain [150,151]. Tissue repair is typically associated with acute or chronic injuries, degenerative diseases, and metabolic disorders. BAPPs function as effective therapeutic agents for tissue regeneration, providing specific benefits. BAPPs, in contrast to other bioactive compounds like small-molecule drugs, nucleic acids, ions, nanoparticles, and micro/nanostructures, specifically target and interact with receptors, exhibiting limited side effects and possessing highly complex functions [76,152]. BAPPs demonstrate inherent biocompatibility and biodegradability, attributed to their natural occurrence in the tissue microenvironment where they participate in biological processes. BAPPs possess attributes that allow them to influence the microenvironment through ROS, blood and lymphatic vessels, immune cells, and repair cells, facilitating tailored tissue repair at designated anatomical locations [77]. BAPPs perform various functions to influence complex microenvironments for tissue regeneration; however, their therapeutic efficacy is constrained mainly by their short half-life and vulnerability to enzymatic degradation. The effect of BAPPs is closely associated with loading concentration and release kinetics. Consequently, novel delivery platforms have been created to integrate BAPPs, maintaining their bioactivity and protecting them from enzymatic degradation. Modifying these delivery systems can enhance loading efficiency and facilitate rapid, controlled, sustained, or heterogeneous release. The strategic integration of various delivery platforms facilitates the sequential deployment of multiple BAPPs for tissue repair [152]. These approaches may reduce healing time and support tissue regeneration (Level 2), but there is limited evidence that such improvements consistently translate into reduced scarring or improved patient satisfaction (Level 1).

In summary, bioactive scaffolds composed of natural biopolymers exhibit promising regenerative effects at the cellular and tissue levels. However, their capacity to solve defined clinical problems—such as minimizing hypertrophic scars, improving mobility, or reducing the need for secondary interventions—requires further validation in context-specific, outcome-driven studies. Bridging the gap between molecular activity and clinical utility remains a key direction for future research.

## 5. Cellular Mechanisms of Natural Products in Scar-Free Healing

Natural products exhibit potential in wound healing through antioxidant, anti-inflammatory, and antimicrobial mechanisms [107]. This section briefly outlines the significance of these characteristics in the wound healing process and their interrelationship, mainly focusing on Level 3 outcomes of the hierarchical framework.

### 5.1. Regulation of Inflammation

Regulating the inflammatory phase is a crucial therapeutic approach in wound treatment since prolonged or severe inflammation may result in delayed healing or chronic wounds. Numerous natural compounds have anti-inflammatory characteristics by means of cytokine suppression, immune cell modulation, and the inhibition of proinflammatory signaling pathways [110]. Table 6 [110,119,153,154] delineates several natural substances and their functions in promoting inflammation resolution and augmenting tissue healing [110].

Nitric oxide production also indicates the inflammatory status of macrophages. Research shows that catechins inhibit nitric oxide release in a dose-dependent manner within a concentration range of 10–40 μg/mL and exhibit anti-inflammatory effects on RAW264.7 macrophages. Combining quercetin and catechin resulted in a 78% reduction in TNF-α and a 75% reduction in IL-1β levels relative to the control group. The observed reduction was significantly greater than that achieved with individual compounds, suggesting their synergistic anti-inflammatory effects associated with NF-κB inhibition in macrophages. Flavonoids selectively modulate inflammation; for example, EGCG is suggested to have a proinflammatory effect at low levels of inflammatory markers and a counterinflammatory effect as these markers rise. These efficacies correspond more closely with the various stages of wound healing and help reduce scarring (Level 2). Nonetheless, more in-depth studies should be performed to fully assess their potential at Level 1—clinical outcomes.

### 5.2. Balancing Oxidative Stress

Increased ROS levels at the wound site hinder cellular proliferation, damage proteins and nucleic acids, trigger apoptosis, and prolong the inflammatory response, thus promoting scarring [155,156,157]. Furthermore, increased free radical levels result in the conversion of fibroblasts to the adherent type, which promotes scarring via heightened collagen synthesis and deposition. Antioxidant enzymes can be activated in physiological conditions, neutralizing free radicals and diminishing ROS [155]. Natural substances alleviate oxidative stress during wound healing by regulating ROS levels and enhancing intrinsic antioxidant defenses. In the first inflammatory phase, elevated ROS are mitigated as several phytochemicals stimulate the nuclear factor erythroid 2-related factor 2 (Nrf2) pathway, enhancing the expression of cytoprotective enzymes such as heme oxygenase-1 (HO^−1^) and superoxide dismutase (SOD) [102]. The xanthophyll astaxanthin not only directly neutralizes free radicals but also induces Nrf2-mediated elevations in HO-1 and SOD, therefore protecting mitochondria from oxidative damage. Astaxanthin mitigated ROS-induced inflammation and averted mitochondria-associated apoptotic damage in a burn wound model. Likewise, the flavonoid baicalin mitigates ROS-induced inflammation by obstructing NF-κB signaling and stimulating Nrf2/HO^−1^, thus reinstating antioxidant enzyme levels [102]. Baicalin’s antioxidant properties reduce macrophage-derived ROS and facilitate macrophage polarization towards the prohealing M2 phenotype, hence expediting the resolution of inflammation in diabetic wounds. In the proliferation phase, natural antioxidants sustain an ideal redox environment for tissue regeneration. Silibinin, derived from *Silybum marianum*, enhances fibroblast resistance to oxidative stress by greatly augmenting intracellular antioxidant capacity and safeguarding fibroblasts from H₂O₂-induced damage, thereby maintaining cell viability for collagen production and angiogenesis [158]. Wounds treated with astaxanthin consistently exhibit elevated levels of collagen I and bFGF expression, indicating enhanced granulation and neovascularization. During the remodeling phase, maintained redox equilibrium inhibits abnormal tissue breakdown. Astaxanthin inhibits ROS-activated matrix metalloproteinases (MMP-1, -3), which would otherwise break down collagen, hence aiding in the preservation of extracellular matrix integrity. Diverse antioxidants, including astaxanthin, baicalin, and silibinin, together maintain a balanced ROS signaling environment via Nrf2/HO^−1^ activation and associated pathways, so safeguarding mitochondrial function and facilitating orderly inflammation, proliferation, and remodeling of wounds [159].

### 5.3. Antibacterial

Antibacterial agents are compounds that either inhibit bacterial growth or eliminate bacteria entirely. These are frequently utilized in diverse applications, such as medical therapies and hygiene products. Wound infection complicates the healing of skin defects, and the emergence of drug-resistant bacteria poses challenges for wound debridement and the effectiveness of antibacterial agents [114,160]. *Escherichia coli* and *Staphylococcus aureus* are common bacteria linked to wound infections. The metabolic byproducts of these bacteria promote inflammation and exudation; for example, the overproduction of MMPs attracts more inflammatory neutrophils to the wound, obstructing the growth and migration of cells in the basal skin layer. A substantial influx of fibroblasts aids in defect repair and the development of proliferative scars. The bacterial metabolite lipopolysaccharide (LPS) markedly diminishes local macrophage recruitment to the wound, suppresses collagen deposition, and elevates apoptotic cell levels in the dermis and granulation tissue at the wound margins. This results in a persistent inflammatory response and extended wound healing duration. Contemporary antibacterial agents may influence the physiological functions of normal cells. Silver demonstrates cytotoxicity towards keratinocytes and fibroblasts, potentially hindering wound healing and causing scarring [161]. The substituents on the benzene ring significantly affect the antibacterial efficacy of flavonoids. Hydrophobic substituents, particularly hydroxyl groups, enhance antibacterial activity, while methylation of these groups reduces this effect [162]. Cushnie et al. [163] investigated the antibacterial properties of flavonoids, revealing three mechanisms: inhibition of bacterial nucleic acid synthesis, disruption of cell membrane function, and interference with energy metabolism. Flavonoids inhibit pore proteins in the bacterial outer membrane, directly affecting *E. coli* by disrupting its energy sources, such as glucose and amino acids. Flavonoids provide cellular protection by inhibiting bacterial adherence and reducing bacterial toxin production. They also inhibit bacterial activity directly and exhibit synergistic effects with antibiotics by reducing the expression of β-lactamases, which contribute to antibiotic resistance in bacteria. Quercetin notably reduces extracellular matrix targeting, thus impairing *E. coli* colonic biofilms. Apigenin increases antibiotic susceptibility in drug-resistant bacteria and limits their dissemination by activating the host’s innate immune system. Flavonoids demonstrate antibacterial activity in mildly alkaline environments [114,164,165].

### 5.4. Antifungal

Antifungal agents are crucial due to the rapid colonization of burns and severely injured skin by fungi, which can result in significant wound infections. Fungal cell walls are composed of several carbohydrate layers, notably α-mannan and β-glucan. α-Mannan binds to dendritic-cell-associated C-type lectin-2 (dectin-2), activating the NF-κB pathway and enhancing the production of the inflammatory cytokine TNF-α, while concurrently inhibiting angiogenesis and myofibroblast proliferation [166]. The mycelium of the fungus and its elastic biofilm may impede wound healing. Plant-derived natural flavonoids can induce apoptosis and reduce biofilm formation, resulting in antifungal effects via multitarget mechanisms. Flavonoids inhibit fungal biofilms by targeting the essential enzyme isocitrate lyase (ICL), enabling Candida albicans to survive and proliferate in nutrient-limited environments within phagocytes such as macrophages and neutrophils, resulting in cell shrinkage and leakage of internal components. Flavonoids impede the synthesis pathway of folic acid, thus inhibiting fungal reproduction [164,165]. Navarro-Martinez et al. [167] cocultured EGCG with dihydrofolate reductase from Candida albicans at varying concentrations, establishing an inhibition constant (Ki) of 0.7 M. EGCG appears to inhibit ergosterol production in fungal cell membranes by disrupting folic acid metabolism, thereby enhancing the synergistic effects of azole antifungals. Clinical trials of a rutin-rich plant ointment for canine wound treatment showed significantly improved wound retraction rates compared to controls and notable antifungal activity against Candida krusei in adjacent areas.

### 5.5. Modulating Fibroblast-to-Myofibroblast Transition

The amount of accumulated collagen was associated with the quantity of mobilized fibroblasts. The ratio of En1-positive to En1-negative fibroblasts in skin defects is the main determinant of scarring [168,169]. En1-positive fibroblasts activate in response to inflammation, ROS, bacterial presence, and wound tension. Moreover, the recruitment of deeper dermal fibroblasts, which are larger, exhibit slower proliferation rates, and secrete substantial collagen fibers, takes place in response to deeper wounds under heightened tension while concurrently inhibiting collagen degradation by decreasing cellulase release. The activation of proscar-forming fibroblasts acts as a protective mechanism against infection and promotes rapid recovery; however, excessive extracellular matrix synthesis and deposition may result in scar formation as a final repair outcome. Flavonoids regulate inflammation, ROS, and infection in wound healing, while enhancing healing processes without causing excessive fibrosis and scar formation [170]. Flavonoids have been shown to decrease TGF-β1 and IL-1β production, inhibit extracellular matrix secretion, and reduce excessive fibrous connective tissue deposition. Safranin preserves fibroblast viability, inhibits TNF-α expression, reduces ECM protein synthesis, and promotes wound healing. Pinocembrin, the primary flavonoid in propolis, demonstrates antifibrotic properties by inhibiting TGF-β1 signaling and interfering with TGF-β1-induced proliferation and activation of abnormal skin fibroblasts, thus significantly mitigating bleomycin-induced excessive skin fibrosis. Quercetin treatment in murine wounds showed similar healing duration and rate to the control group but resulted in decreased ECM deposition in vivo [61,63]. Quercetin also affected fibroblast adhesion and migration, reducing scar tissue fibrosis [168].

Overall, even though natural compounds exhibit promising effects across several mechanistic domains, further translational studies are needed to define how modulation of these individual processes contributes to clinically meaningful improvements in scar quality, tissue function, and patient satisfaction. Establishing quantitative links across hierarchical levels remains a central challenge in developing effective, outcome-driven therapies.

## 6. Emerging Innovations in Scar-Free Healing

### 6.1. Micro- and Nanomaterials

Microemulsions (MEs) are homogeneous, single-phase, clear, and thermodynamically stable colloidal mixtures composed of water, oil, cosurfactant, and surfactant, with droplet sizes generally not exceeding 100 nm. While microemulsions exhibit smaller droplet sizes than nanoemulsions (NEs), the terminology for both remains in use [54]. MEs provide a viable platform for drug delivery, enhancing solubility, stability, absorption, and targeted delivery to improve therapeutic outcomes. The synergistic impact of ME-encapsulated curcumin and docosahexaenoic acid on safeguarding WRL-68 cells from high-fat-induced hepatic injury and suppressing LX2 cells has been recorded [171]. The codelivery of these components markedly decreased serum triacylglycerol and low-density lipoprotein cholesterol levels in mice with non-alcoholic fatty liver disease. Micelles exhibit a significant capacity to entrap hydrophobic substances within their internal oily phase, safeguarding against oxidative and enzymatic degradation. The developed MEs showed increased antioxidant activity and enhanced antimicrobial effectiveness against *E. coli* and Gram-positive S. aureus. MEs have proven to be a safe and effective method for curcumin delivery, exhibiting notable cytotoxic and in vivo anti-inflammatory effects. NEs have emerged as effective nanocarriers due to their unique capacity to encapsulate lipophilic and lipophobic substances, offering various possibilities for drug delivery applications [171]. NEs exhibit considerable promise in improving various pharmaceuticals’ pharmacokinetics and clinical efficacy. Medium-chain triglycerides as carrier lipids in emulsions significantly enhance curcumin’s bioaccessibility. NE curcumin has been investigated for its bioaccessibility, wound healing properties, antiarthritic, anti-inflammatory, antifungal, and antiparasitic effects, quorum sensing, antineoplastic potential, cardioprotective benefits, and neurodegenerative impacts [172].

Nanostructures have been engineered to surmount the obstacles linked to free therapies and traverse the biological barriers—microenvironmental, systemic, and cellular—that exhibit variability across patient groups and illnesses. A nanocomposite using *C. asiatica* glycolic extract (2:1 E/D ratio) and fumed SiO_2_ has been formulated as an innovative approach for addressing skin damage. This method utilizes the characteristics of both *C. asiatica* and the silica matrix, which may function as a medication delivery mechanism and improve wound healing. Silica is recognized for facilitating tissue healing via the enhancement of collagen production and angiogenesis. Scano et al. [173] showed that incorporating natural products into silica-based delivery methods safeguards these extracts from physical and chemical degradation, thereby improving their bioavailability and pharmacological efficacy. Employing biomaterials derived from medicinal plant components presents a significant approach to enhancing topical therapy for skin injuries, perhaps obviating the need for systemic treatments [174,175]. In this context, the ball milling procedure was used to integrate the *C. asiatica* glycolic extract into the silica pores while preserving the extract’s characteristics. The resultant nanocomposites demonstrated significant antioxidant activity, superior biocompatibility, and protective effects on cells against hydrogen peroxide. Furthermore, the incorporation of cosmeceuticals with nanotechnology is a method that may provide significant advantages, including enhanced skin absorption, increased physical stability, and controlled release of active ingredients [174].

Niosomes, a nanoscale carrier system, encapsulate hydrophilic and lipophilic chemicals inside vesicles. These carriers are both biodegradable and biocompatible. Topical formulations containing niosomes have shown increased drug accumulation in the deeper layers of the epidermis and dermis. Niosomes may be surface-modified with suitable polymers to enhance the permeability of hydrophilic substances into deeper skin layers. Hyaluronic acid (HA), a natural constituent of healthy skin tissue, serves as a penetration booster. To achieve targeted distribution of asiaticoside to the deeper layers of the skin, *C. asiatica* extract-loaded niosomes (CAE-Nios) and niosomes modified with hyaluronic acid (CAE-Nio-HAs) were formulated (specifics of the *C. asiatica* extract not provided). Wichayapreechar et al. [175] discovered that niosomes exhibited limited systemic permeability while facilitating increased drug penetration and accumulation in the dermal layer of the skin. The surface modification with HA enhanced the transdermal absorption of hydrophilic substances in CAE via pig skin. Hyaluronic acid has a strong affinity for the polysaccharides inside the stratum corneum, facilitating its absorption into the outer skin layer and improving medication retention in the dermis. Consequently, HA-modified niosomes provide a viable method for the delivery of polar pharmaceutical agents such as CAE into the deeper layers of the skin, hence enhancing topical medicinal compositions [175,176].

In summary, micro- and nano-engineered materials offer enhanced drug stability, penetration, and targeted delivery, modulating inflammation, angiogenesis, and ECM remodeling (Level 3). These technologies show strong potential to accelerate re-epithelialization and reduce infection risk (Level 2). However, whether such improvements result in reduced scar revision rates, functional recovery, or improved aesthetic outcomes (Level 1) remains largely unexplored in controlled clinical trials.

### 6.2. Composite Materials

**Figure 8:**
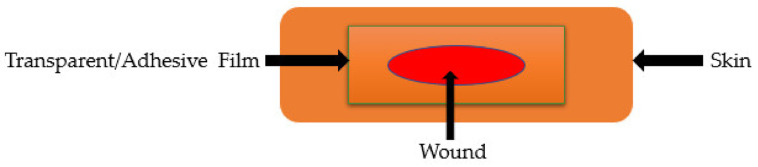
Film Dressing on Skin Wound. Reprinted from an open-access source [142].

Films are semi-permeable dressings characterized by their translucent and adhesive properties [142], as can be observed in Figure 8. They create a humid environment, facilitate cell migration, encourage autolysis, exhibit partial water vapor and oxygen permeability, and suppress bacterial growth. Films are appropriate for managing chronic wounds on the heels, elbows, and flat body surfaces and moderate exuding and superficial wounds. These dressings are economical, provide clarity regarding necrotic debris, are impermeable to water, and allow for repeated evaluations of a wound (Level 1). They have the drawback of inducing skin maceration upon removal, and many are either non-absorptive or exhibit low absorption capabilities [18,177].

Numerous researchers have reported on gelatin-based hybrid film wound dressings. Taheri et al. [178] developed gelatin–chitosan hybrid films containing tannic acid and/or bacterial nanocellulose for wound healing purposes. In vivo studies with Wistar rats indicated that full-thickness skin wounds treated with dual drug-loaded and nanoparticle-loaded films exhibited faster healing than tannic-acid-loaded and plain hybrid films. Sakthiguru and Sithique [179] developed gelatin–chitosan biocomposite films infused with allantoin for wound healing purposes. The water absorption tests indicated improved absorption capacity in allantoin-incorporated hybrid films. In vitro cytotoxicity tests of all films, evaluated via the MTT assay, showed over 80% cell viability with L929 fibroblasts, indicating strong biocompatibility and non-toxicity. Antimicrobial studies indicated that allantoin-loaded films exhibited enhanced antibacterial activity against *S. aureus* and *E. coli* compared to free allantoin and plain films, suggesting that allantoin-incorporated dressings may serve as effective antibacterial wound dressing materials.

Akhavan-Kharazian and Izadi-Vasafi [180] synthesized hybrid films composed of gelatin and chitosan. The films incorporated nanocrystalline cellulose and calcium peroxide for wound healing. The swelling analysis indicated significant swelling at pH levels 5, 7, and 9, corresponding to wound exudate, physiological conditions, and infected wounds, respectively. The hybrid films’ water vapor permeation studies revealed WVTR values between 35 and 45 g/m^2^/h, suitable for maintaining optimal fluid balance on the wound bed. The in vitro cytotoxicity analysis via the MTT assay demonstrated nearly 100% cell viability of human fibroblast cells immersed in dual-loaded and plain films for seven days, indicating that the gelatin-based hybrid films are non-toxic. Patel et al. [181] developed gelatin–chitosan films for the drug delivery of lupeol in wound dressing applications. The SEM micrographs of the hybrid films exhibited a smooth, fibrous, and porous morphology conducive to enhancing oxygen supply to the injury, thereby facilitating accelerated wound healing.

Various types of wound dressings can be created by combining gelatin with different polymers, as illustrated in Table 7. All in all, the combination of natural bioactives with synthetic scaffolds enables synergistic effects on cell adhesion, antimicrobial activity, and cytokine modulation. These advances represent a promising avenue to optimize the wound microenvironment (Level 3) and healing dynamics (Level 2). Yet, their impact on resolving specific clinical problems—such as postsurgical scar minimization or restoring dermal elasticity—has not been definitively shown (Level 1), highlighting the need for outcome-driven evaluations.

### 6.3. Smart Dressings

Smart materials capable of responding to wound stimuli (e.g., pH, temperature) introduce personalized modulation of healing factors (Level 3). Early studies suggest they can reduce healing time and limit infection-related delays (Level 2). Nevertheless, whether this dynamic control results in fewer complications or improved long-term cosmetic and functional results remains to be substantiated in comparative clinical studies (Level 1).

**Figure 9:**
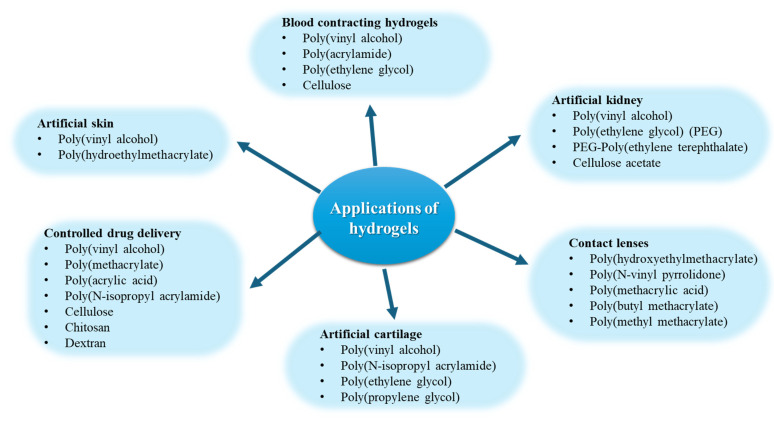
Applications of hydrogels in different biomedical fields. Created based on information from [186,187,188].

pH-sensitive hydrogels have been utilized in biomedical applications such as targeted oral drug delivery, leveraging the gastrointestinal tract’s pH gradients, and biosensors, including microdevices that employ pH-responsive poly(methacrylic acid)-PEG films. Figure 9 summarizes the applications of hydrogels across various biomedical fields. Wound healing also utilizes these “smart” materials. Wound environments experience notable pH variations: healthy skin maintains a mildly acidic pH of approximately 5.0–6.0, acute wounds shift to near-neutral levels around 7.4, and chronic or infected wounds frequently become alkaline, ranging from pH 7.4 to 9.0. The changes in wound pH are associated with healing status; for instance, chronic non-healing wounds typically maintain an alkaline environment, providing a basis for targeted therapies [183]. pH-responsive hydrogels are investigated as intelligent wound dressings capable of sensing and adapting to variations in wound pH, enabling on-demand drug release or real-time signaling of changes. Many of these dressings are composed of natural polymers and bioactive compounds, essential for ensuring biocompatibility and bioactivity in wound care. Polysaccharides such as alginate, hyaluronic acid, and chitosan possess pH-responsive functional groups that can be acidic or basic. Their swelling or shrinking behavior, influenced by protonation, adjusts the hydrogel’s pore size and drug release characteristics. Researchers have developed smart dressings using naturally derived polymers, often combined with natural bioactive agents, that maintain a moist, physiologically favorable environment and respond to infection-related pH changes. Literature studies examined recent developments in pH-sensitive wound dressings that utilize natural product components, focusing on their roles in facilitating controlled drug release, infection detection, and promoting scar-free tissue regeneration over the past 5 to 10 years [183,184].

Chronic wounds and burns often necessitate continuous administration of antimicrobials, anti-inflammatories, or growth factors. pH-sensitive natural polymer hydrogels act as reservoirs that release therapeutic agents more quickly in the elevated pH of infected or chronic wounds while retaining them at pH levels closer to normal skin [183]. A recent study [185] created a hydrogel using locust bean gum grafted with poly(acrylamide-co-acrylic acid), a chemically modified natural polysaccharide that is pH-responsive and incorporated the natural antioxidant protein C-phycocyanin. The dressing showed limited drug release at acidic pH, while significantly higher release (~64% of payload over 48 h) occurred at neutral pH (7.4). In diabetic rat wound models, the pH-tuned release of C-phycocyanin accelerated wound closure and significantly decreased inflammatory cytokines (IL-6, IL-1β, TNF-α) in the wound tissue, suggesting reduced inflammation and improved healing. A notable system is a composite hydrogel that integrates bacterial cellulose, a natural biopolymer, with a minor quantity of polyvinyl alcohol and graphene oxide for stabilization. This hydrogel incorporated the natural polyphenol curcumin, derived from turmeric, as an antimicrobial agent [185]. The dressing maintained its integrity in mildly acidic conditions but expanded and deteriorated at approximately neutral pH levels of wound exudate, releasing curcumin in a controlled fashion. In vitro tests demonstrated pH-dependent kinetics: drug release peaked at pH 7.4 (exceeding 90% in 33 h) and was markedly reduced at pH 6.4 and 8.4. This on-demand delivery can enhance antimicrobial or anti-inflammatory effects during infection peaks and decrease as pH normalizes, preventing the need for constant high dosing. The authors observed that these pH-responsive bacterial cellulose hydrogels exhibited inherent biocompatibility, robust mechanical integrity, and broad-spectrum antimicrobial activity, indicating their potential for burn and chronic wound care. Researchers have utilized innovative chemistry to accomplish dual functions. In a specific design, tobramycin, an aminoglycoside antibiotic, served as a crosslinking agent to construct a natural hydrogel network. In this study, dialdehyde-modified carboxymethyl cellulose (derived from cellulose fiber) was crosslinked through Schiff-base bonds with the amine groups of tobramycin, while β-cyclodextrin (a cyclic natural sugar) was incorporated to encapsulate a second therapeutic agent (an antioxidant). The outcome was a biopolymer hydrogel containing tobramycin: as the wound environment becomes more acidic, which can happen in bacteria-laden biofilms or specific healing phases, the imine bonds slowly hydrolyze, releasing tobramycin locally. This dual-drug, pH-responsive hydrogel demonstrated significant antibacterial activity from tobramycin and antioxidant properties from the second drug, highlighting the potential of combining natural-product-based components for pH-triggered multifaceted therapy [183,184,185].

### 6.4. Injectable Hydrogels

Injectable hydrogels have gained importance for the localized and sustained delivery of drugs in a single administration. This will reduce side effects and systemic drug concentration. Ischemic regions, tumors, and sites of wound healing exhibit acidity. Dual pH- and temperature-sensitive hydrogels have garnered attention from researchers for the development of drug delivery systems aimed at targeting areas of local acidosis. Drug-carrying pH-responsive hydrogels must exhibit swelling in areas of local acidosis (pH < 7 down to 5) to facilitate controlled drug release while demonstrating minimal swelling at physiological pH (7.40). Garbern et al. developed pH-sensitive hydrogels made from a copolymer of N-isopropylacrylamide and propylacrylic acid for the controlled release of growth factors as injectable hydrogels [189,190]. These hydrogels can release the growth factor over three weeks. The ionic nature of injectable dual pH/temperature-sensitive hydrogels is a critical characteristic, facilitating the formation of ionic complexes with various bioactive species or factors. Positively charged hydrogels can form ionic complexes with anionic species such as insulin, while negatively charged hydrogels interact with cationic species like transforming growth factor beta. Nguyen et al. [191] developed a new class of amphoteric hydrogels, specifically a poly(urethane-amino-sulfamethazine)-based block copolymer, capable of forming cationic and anionic hydrogels in response to acidic and basic pH conditions, respectively. The aqueous solution of these copolymers forms a gel in the pH range of 6.8 to 8.2 at elevated temperatures while exhibiting a free-flowing sol in slightly acidic and basic conditions. The amphoteric hydrogels were evaluated for the release of anionic protein and human growth hormone (hGH). The release was effectively regulated over three days postinjection, exhibiting minimal initial burst release in the serum of Sprague Dawley rats compared to hGH solution. The controlled release of hGH from these amphoteric hydrogels results from the formation of ionic complexes between the cationic components of the hydrogels and the negatively charged protein.

Thus, injectable hydrogels serve as minimally invasive vehicles for delivering bioactives directly into complex wound geometries, facilitating ECM mimicry and sustained release (Level 3). These materials may shorten healing times or reduce fibrosis (Level 2), but their clinical efficacy in preventing hypertrophic scars, contractures, or need for revision has yet to be demonstrated conclusively (Level 1).

Table 8 emphasizes novel biomaterial advancements aimed at promoting scar-free healing, concentrating on intelligent dressings and injectable hydrogel systems. These systems include natural compounds like curcumin, resveratrol, and therapeutic peptides into synthetic matrices that exhibit responsive behaviors, including thermosensitivity, reactive oxygen species scavenging, and prolonged bioactive release. The table presents preclinical and early-stage clinical instances, comparing natural-product-based technologies with significant synthetic systems to demonstrate their respective therapeutic efficacy. Collectively, these formulations signify the advancement of wound therapeutics that integrate bioactivity with adjustable administration and mechanical characteristics designed for regenerative results.

While emerging innovations in wound biomaterials hold considerable promise for enhancing biological repair processes, their ability to solve defined clinical problems (i.e., restoring tissue mechanics, minimizing long-term scarring, or improving patient satisfaction) requires further validation through application-specific, outcome-focused studies.

## 7. Clinical Applications and Case Studies

The healing of wounds, particularly chronic or complex ones, continues to pose a substantial therapeutic challenge. Synthetic therapies such as antibiotics and growth hormones are conventional; nonetheless, apprehensions about adverse effects, antibiotic resistance, and expenses have spurred interest in natural alternatives. This section presents current clinical research on natural-product-based therapy for wound healing, including diverse wound types such as diabetic ulcers, surgical wounds, and burns.

### 7.1. Patches Based on Natural Products

Traditionally, *C. asiatica* extract is delivered by ointments or creams. Nevertheless, the outermost epidermal layer exhibits a degree of impermeability to lipophilic compounds such as asiatic acid, potentially restricting the efficacy of these traditional formulations. This difficulty may be mitigated with sophisticated medication delivery methods. The PerioPatch^®^ (MIS Implants Technologies Ltd., Misgav, Israel) is a medicinal patch that comprises extracts from *C. asiatica*, *Echinacea purpurea*, and *Sambucus nigra*. This medical gadget has shown substantial therapeutic benefits, especially in wound healing and collagen production, and may substantially reduce gingival inflammation. Chaushu et al.’s research [200] demonstrated that the PerioPatch^®^ improved gingival healing in comparison to control and placebo patches. This indicates that active herbal constituents are significant in facilitating healing, as seen by increased cell proliferation [176,200]. These findings are especially relevant for caring for gingival wounds, solving the problem of associated discomfort through accelerated healing and reduced inflammation (Level 2).

### 7.2. Natural Treatments for Diabetic Foot Ulcers

Diabetic ulcers are characterized by chronic inflammation and delayed re-epithelialization, increasing the risk of infection and amputation and requiring advanced alternatives for specialized wound care [201]. In this respect, various natural products have been explored, with effects being noticed at all three hierarchical levels of wound management: Level 3—biological mechanisms, Level 2—healing dynamics, Level 1—clinical outcomes.

Papaya latex enzymes (Protease P1G10): An innovative therapy for diabetic foot ulcers is a proteolytic extract from *Vasconcellea cundinamarcensis* latex, referred to as P1G10. A double-blind Phase II trial involving 50 patients compared a 0.1% P1G10 ointment to standard hydrogel dressing [202]. The papaya enzyme treatment notably enhanced healing outcomes, with a greater proportion of patients attaining complete (100%) wound closure in a reduced timeframe compared to controls. In the P1G10 group, 11 patients achieved full closure compared to 5 in the control group, and a higher overall proportion attained ≥80% closure, with no adverse effects noted. The protease functions as a debriding agent, facilitating the removal of necrotic tissue and potentially disrupting bacterial biofilms, thus promoting the healing process (Level 2). This result indicates that papaya-derived enzymes may serve as a safe and effective adjunct for diabetic ulcers, subject to further large-scale trials [202,203].

Marigold (*Calendula officinalis*) extract: Calendula has a historical significance in wound care, with a 2016 prospective study investigating a hydroglycolic extract of *Calendula officinalis* for diabetic foot ulcers [202,203]. All 41 patients were administered topical calendula extract, with 78% of ulcers achieving complete closure within 30 weeks (mean healing time approximately 15.5 weeks). Patients exhibited a notable decrease in bacterial colonization (Level 2) and reported alleviated pain following the calendula treatment (Level 1). This open-label pilot study offers real-world evidence of calendula’s wound healing potential, linked to triterpenoid compounds with anti-inflammatory properties, highlighting the necessity for controlled trials. Calendula ointments are utilized as low-risk treatments to enhance granulation and epithelialization in chronic foot wounds (Level 2) [202].

Fish skin grafts: In addition to botanicals, biologic dressings derived from marine sources are now in clinical use. A skin graft from North Atlantic cod fish skin, which is high in omega-3 and collagen, has demonstrated significant efficacy in treating diabetic foot ulcers involving bone or tendon exposure. A recent international RCT, the “ODINN” trial, involved 255 patients with deep diabetic ulcers who were randomized to receive weekly applications of fish skin graft or standard care. At 16 weeks, 44% of patients in the fish skin group experienced complete healing, in contrast to 26% in the standard care group (*p* < 0.001). At 24 weeks, the disparity remained (55% vs. 38% healed), with a significantly quicker healing time associated with fish skin (hazard ratio ~1.59) (Level 2) [204]. Wound infections were observed in both groups, with a marginally higher incidence in the fish skin arm; however, the overall advantage in healing rates is evident. The findings suggest that intact fish skin xenografts function effectively as regenerative scaffolds for challenging diabetic ulcers, demonstrating the successful application of a natural marine product in advanced wound care [202,204].

Honey dressings: Honey-based wound care products may aid in infection management by diminishing the pathogen count to a level that allows the host to defend itself or by lowering the pathogen levels to prevent crossinfection. Honey dressings should be used until the wound exhibits indications of healing, after which they may be withdrawn [205]. Antimicrobial dressings should be used for no more than four weeks if no progress is seen. A comprehensive re-evaluation should be conducted to ascertain variables that may contribute to protracted healing. Addressing the fundamental molecular and cellular imbalance that may contribute to indolence is increasingly acknowledged as crucial for chronic wound management in primary care settings. The literature review revealed just two qualifying papers, highlighting a lack of randomized controlled trials comparing honey with hydrogel for wound management [206,207]. Ingle et al. [208] conducted a study in South Africa with 82 participants, using randomization and blinding techniques. The inclusion of HIV-positive individuals may have affected results [209] since previous research suggests a greater prevalence of wound dehiscence in this demographic. Nevertheless, the baseline features of the honey and hydrogel groups were comparable. Al Saeed et al. [210] performed research with 71 patients suffering from diabetic foot ulcers, evaluating the efficacy of manuka honey (MH) vs. silver hydrogel dressings. The honey group required daily dressing changes, whereas hydrogel could be sustained for up to seven days. The lack of formal blinding compromised the study’s validity. The honey group exhibited a shorter healing time than hydrogel. However, this difference lacked statistical significance (*p* > 0.05). Ingle et al. [208] noted similar results for superficial wounds; however, hydrogel has shown superior effectiveness in abrasion wounds. Ingle et al. [208] performed an examination of slough and necrotic tissue, demonstrating a greater prevalence of slough in the honey group. Nonetheless, healing accelerated, presumably due to the antibacterial characteristics of honey derived from hydrogen peroxide synthesis. The antibacterial effectiveness of honey is determined by the nectar source [206,207,209]. While these are positive indicators, the studies did not report whether these improvements led to reductions in amputation risk or recurrence rates (Level 1 outcomes).

### 7.3. Natural Products in Venous Leg Ulcers

Chronic venous leg ulcers represent a domain where clinical exploration of natural remedies has occurred. One example is the extract from mimosa tree bark. The bark of *Mimosa tenuiflora*, or tepezcohuite, recognized in traditional medicine, was developed into a hydrogel for treating venous ulcers. A randomized trial involving 40 patients demonstrated that those receiving the mimosa extract gel experienced a reduction in ulcer area and achieved wound closure, while the placebo group exhibited worsening ulcers (increased ulcer area) [203]. A subsequent study indicated that ulcers treated with mimosa exhibited a greater rate of re-epithelialization (58% compared to 39% in controls) (Level 2), though the difference in final ulcer size was not statistically significant in the smaller sample (Level 1) [204]. Clinical studies validate the anecdotal use of mimosa bark in wound healing, linking its efficacy to antimicrobial properties and stimulation of fibroblast activity [203].

A pilot trial assessed a combined herbal therapy for venous ulcers. This study involved 17 patients who were treated with Plantoderm^®^ ointment, which contains extracts of *Calendula officinalis*, *Symphytum officinale*, *Achillea millefolium*, and *Salvia officinalis*, applied to the ulcer. Additionally, Fitoven^®^ gel, containing *Aesculus hippocastanum*, *Melilotus*, *Rosmarinus*, and *Lavandula* extracts, was massaged into the surrounding skin [211]. A control group of 17 patients underwent standard wound care, which included cleansing, dressings, and antibiotics as necessary [212]. After 7 weeks, the herbal-treatment group exhibited a ~42.7% reduction in ulcer area, significantly surpassing the ~35.6% reduction observed in the control group. The herbal group exhibited a more significant reduction in bacterial load in wounds, with four patients’ ulcers becoming completely culture-negative, indicating an antiseptic effect (Level 3). Patients exhibited good tolerance to the herbal ointments, and the multiextract formulation seemed to enhance healing with minimal side effects. Although limited in size, this 2010 study [153] demonstrates that botanical topical therapy can enhance venous ulcer outcomes compared to standard care.

### 7.4. Applications of Natural Products in Burn Wounds

Burn wounds often result in extensive fibrosis and contracture, limiting range of motion and necessitating surgical revision [188]. Burn care has a significant history of natural therapies, with recent trials reinforcing the efficacy of specific traditional remedies.

*Aloe vera* is often referenced for its efficacy in healing burn wounds [213]. A 2022 randomized clinical trial evaluated the efficacy of topical Aloe vera gel versus 1% silver sulfadiazine (SSD) cream in patients with first- and second-degree burns. Thirty-four patients received treatment with aloe gel dressings, while another 34 were treated with SSD. Both groups attained complete wound epithelialization within two weeks, though the aloe group exhibited a more rapid healing process (Level 2). Patients treated with aloe reported reduced pain during healing and immediate itch relief: on day 7, aloe gel application significantly alleviated pruritus within 30 min, while the control group experienced prolonged itching (Level 1). By day 14, no patients treated with aloe experienced residual pain, akin to the SSD group, yet overall pain scores were reduced with aloe [211]. The findings, supported by previous meta-analyses, demonstrate that *Aloe vera* accelerates burn wound epithelialization and enhances patient comfort, reducing itch and pain compared to standard antimicrobial cream. The anti-inflammatory and hydrating properties (Level 3) of aloe likely contribute to its effectiveness as a natural remedy for mild to moderate burns.

Innovations such as fish skin extend beyond diabetic ulcers; they are also utilized for burns. Processed and glycerol-preserved tilapia skin has been utilized as a biological dressing for partial-thickness burns. A Phase III RCT conducted in Brazil from 2017 to 2018 involved 115 burn patients and compared the efficacy of tilapia skin grafts to daily SSD cream. The tilapia skin, abundant in collagen, was applied once and remained in place, while SSD necessitated regular changes [212]. The fish skin treatment notably accelerated wound closure (Level 2), with an average re-epithelialization time of approximately 9.7 days compared to 10.2 days for SSD (*p* = 0.001). Patients required significantly fewer dressing changes (mean ~1.6 with fish skin compared to 4.9 with SSD) and experienced notably lower pain scores and analgesic requirements (Level 1) [212]. The tilapia skin served as a supportive bioscaffold, minimizing trauma during dressing changes and preserving a moist, sterile wound environment. The total treatment cost per patient in the tilapia group was 42% lower, attributed to reduced supplies and decreased nursing time. Clinical outcomes align with real-world findings that cost-effective tilapia skin bandages promote faster and more comfortable healing of burns compared to conventional dressings. This method has gained recognition as an efficient therapy for burn treatment, particularly in resource-limited environments where traditional artificial skin or costly dressings are unavailable.

### 7.5. Natural and Essential Oils in Surgical Wound Care

Surgical incisions can result in hypertrophic scarring, discoloration, or pain, necessitating adequate attention. Natural product applications have proven beneficial for postsurgical wounds, including clean incisions and obstetric wounds, primarily for pain reduction (Level 1) and infection prevention (Level 3).

Lavender oil is a well-researched example for promoting perineal wound healing following an episiotomy during childbirth. Lavender essential oil possesses antiseptic and analgesic properties, with several clinical trials assessing its effects in postpartum women [214]. A systematic review of five RCTs indicated that diluted lavender oil, used as a sitz bath or spray, significantly enhances episiotomy wound outcomes. In three trials assessing healing, lavender treatment demonstrated quicker wound closure and improved Redness, Edema, Ecchymosis, Discharge, Approximation (REEDA) scores compared to standard care (Level 2). All five trials indicated that lavender significantly alleviated pain, leading to lower perineal pain scores and reduced discomfort in the postpartum period (Level 1). A study indicated that by days 5 to 7 postpartum, women using lavender-oil sitz baths experienced significantly reduced pain and less redness and swelling at the incision compared to those using povidone–iodine or a placebo. No adverse effects were reported for either the mother or infant in these studies. The review concluded that lavender essential oil is a safe and effective adjunct for episiotomy wound healing and pain relief. This has implications for other surgical wounds; the anti-inflammatory properties of specific essential oils, such as lavender and tea tree, are being investigated for their potential to alleviate surgical site pain and support minor wound healing. Aromatherapy and topical oils emerge as accessible, cost-effective alternatives for improving postoperative wound care, although further research in varied surgical populations is necessary [215,216].

Table 9 synthesizes recent human studies in which plant extracts, oils, or biologic dressings were tested for their effect on wound repair. Each entry enumerates the natural product or formulation examined, the study design and patient demographic (e.g., randomized controlled trial, pilot study, subject count, and wound classification), the outcome metrics evaluated (including wound closure rate, healing duration, pain or inflammation scores), and the principal conclusions. The table underscores the clinical efficacy of chosen natural medicines and enhances this section’s emphasis on the clinical applications of natural products. By integrating mechanistic ideas with patient-derived information, it highlights the thorough evaluation of traditional treatments in practice, thereby informing safer and more effective wound care practices.

## 8. Challenges and Limitations

Despite the significant potential of natural products for scar-free wound healing, their practical use is hindered by many pharmacological, manufacturing, and regulatory constraints. A primary difficulty is inadequate bioavailability, impacting substances like curcumin, EGCG, and quercetin. Although curcumin has significant anti-inflammatory and antioxidant effects, its therapeutic efficacy is limited by poor water solubility, fast metabolism, instability at physiological pH, and systemic excretion as pharmacologically inactive conjugates [220,221]. EGCG, although beneficial when applied topically, demonstrates instability in alkaline conditions and has low absorption when taken orally. Likewise, quercetin has inadequate water solubility and degradation, necessitating the creation of nanoformulations to enhance tissue permeability and stability. The intricacies of extraction and formulation, including pharmacokinetics, provide substantial obstacles to large-scale manufacturing. Natural substances including propolis, *Centella asiatica*, and ginseng often exhibit variations in bioactive content attributable to regional, seasonal, and agricultural factors [222,223,224]. This variance affects standardization, impedes batch reproducibility, and raises issues over the uniformity of treatment effects. Moreover, extraction techniques—particularly for heat-sensitive polyphenols and triterpenes—must be meticulously calibrated to prevent deterioration or loss of efficacy, hence necessitating expensive and precise technology.

From a technical standpoint, shifting to sustainable manufacturing techniques for plant-based goods imposes cost burdens. Eco-friendly extraction and purification techniques sometimes need innovative infrastructure, resulting in increased initial expenses and extended development periods. This is particularly crucial for chemicals such as resveratrol or baicalin, which need high-purity extraction from intricate matrices. Stringent preclinical and clinical validation is necessary to guarantee safety and effectiveness. Natural products often include intricate combinations including several active constituents, hindering the identification of particular processes or the evaluation of toxicity profiles [223,225,226]. Clinical studies incorporating compounds such as andrographolide, allicin, or astaxanthin need rigorous safety monitoring, standardized doses, and reproducibility—elements that are challenging to regulate in diverse natural extracts. Furthermore, several natural compounds remain devoid of thorough absorption, distribution, metabolism, excretion, toxicity (ADMET) profiles, hindering their approval for therapeutic use. Finally, whereas several natural compounds have multitargeted effects—regulating inflammation, oxidative stress, microbial defense, and fibroblast activity—this diversity may result in unexpected biological consequences if not properly dosed or administered. For instance, whereas flavonoids’ inhibition of TGF-β may diminish fibrosis, excessive suppression might hinder normal ECM remodeling and postpone wound healing. Such hazards need more accurate delivery methods and enhanced mechanistic understanding [156].

Although natural products like curcumin, EGCG, quercetin, and baicalin exhibit considerable promise for enhancing wound healing and reducing fibrosis, their transition into clinical application necessitates addressing challenges associated with bioavailability, extraction complexity, consistency, regulatory compliance, and safety [157]. Overcoming these restrictions via innovative delivery mechanisms (e.g., hydrogels, nanocarriers), sustainable manufacturing advancements, and rigorous clinical validation will be crucial for converting their therapeutic potential into viable and scalable wound care treatments [227].

In addition to regulatory, safety, and scale-up concerns, a critical barrier to clinical translation lies in the limited understanding of how modulation of biological processes (Level 3) translates into acceptable outcomes (Level 1). Across most applications (e.g., burns, diabetic foot ulcers, venous leg ulcers, and surgical wounds) quantitative design constraints are not yet established. For example, it is unclear how much suppression of fibroblast-to-myofibroblast transition is required to prevent contracture in burns or how much acceleration of re-epithelialization is needed to avoid complications in chronic ulcers. Moreover, the relationship between early-stage markers (e.g., cytokine levels, collagen alignment) and long-term functional or aesthetic outcomes remains poorly defined. These knowledge gaps hinder rational design and benchmarking of natural-product-based therapies and highlight the need for translational research that directly links molecular changes to clinical success criteria.

## 9. Future Directions

Bioprospecting offers considerable potential for discovering compounds with scar-inhibiting characteristics. Marine habitats, especially in harsh conditions like the Antarctic, are abundant in distinctive microbes that can synthesize such chemicals. Advancements in next-generation sequencing have improved our capacity to access and assess the biosynthetic potential of these microorganisms, enabling the identification of novel natural compounds with therapeutic uses [228].

Incorporating biomarker-driven approaches into scar-prevention medicines enables treatments customized to specific patient profiles. Predictive biomarkers may identify patients at increased risk of severe scarring, allowing doctors to tailor antifibrotic therapies appropriately. In dermatological disorders such as hidradenitis suppurativa and psoriasis, unique biomarkers have been found that inform tailored therapy, demonstrating the possibility of personalized medicine in scar avoidance [229]. Over the last decade, insights derived from patients’ tissue specimens have identified the first molecular markers linked to healing deficiencies. Tissue from the periphery of non-healing wounds exhibits hyperproliferative epidermis, varying levels of fibrosis, and heightened cellular infiltration [230]. Several research studies have identified several tissue-based molecular markers downstream of the Wnt signaling system, namely nuclear β-catenin and c-myc, which are clinically linked to impaired healing. The Wnt pathway is crucial for skin development and homeostasis throughout early developmental stages, influencing patterning and morphogenesis and playing a role in the postnatal hair cycle, as well as the maintenance and regulation of stem cells and cellular destiny within the epidermal compartments [228]. Laboratory investigations have shown the activation of β-catenin and its downstream target, the oncogene c-myc, in the non-healing margins of chronic wounds, leading to thicker, hyperproliferative, and parakeratotic epidermis, which signifies abnormal proliferation and improper differentiation [9,229,231]. The nuclear presence of these biomarkers in tissue specimens may be quantified using immunohistochemistry, underscoring their clinical feasibility and potential as tissue biomarkers. These two biomarkers are currently receiving further evaluation, and promising early laboratory findings indicate that they may possess considerable predictive and diagnostic capabilities, with clinical validation in progress [232]. Advancements in targeted delivery systems, including microneedles and responsive nanocarriers, have transformed therapeutic approaches. These technologies improve the accuracy and effectiveness of therapies by enabling controlled release and focused administration of bioactive substances [233]. Nanotechnology-based approaches have been devised to treat keloids—elevated scars—more efficiently and safely, demonstrating the promise of sophisticated delivery systems in scar care [230,232].

The integration of natural products with stem cell treatment and tissue engineering shows potential for enhancing soft tissue repair and attaining scar-free results. Bioactive materials, modeled after natural healing mechanisms, have been engineered to target collagen in wounds, facilitating rapid healing and efficient scar prevention. This integrative method utilizes the regenerative potential of stem cells with the structural reinforcement of synthetic tissues, providing a holistic strategy for tissue repair and regeneration. The future directions emphasize a multidisciplinary strategy for achieving scar-free soft tissue repair, including natural product discovery, customized medicine, enhanced delivery technologies, and regenerative medicine [150].

While substantial progress has been made in developing natural-product-based therapies and delivery systems, future efforts must focus on bridging mechanistic advances (Level 3) and improved healing dynamics (Level 2) with validated clinical outcomes (Level 1). This requires new translational studies that measure not only molecular and histological markers but also functional recovery, cosmetic results, revision rates, and patient-reported outcomes. Importantly, application-specific design constraints must be established. Multicenter trials, standardized endpoints, and the integration of clinicians, bioengineers, and regulatory scientists will be essential in transforming promising laboratory findings into clinically effective treatments. Advancing this hierarchy-based approach is critical if natural products are to fulfill their potential in regenerative medicine.

## 10. Conclusions

Skin wound healing is a multifaceted and dynamic process designed to restore barrier function, avert infection, and re-establish mechanical and physiological integrity. Notwithstanding considerable progress in wound healing techniques encompassing growth factor therapy, biomaterial dressings, and cell-based interventions, scar-free soft tissue healing continues to pose significant clinical challenges. The diversity of fibrotic diseases renders single-target therapy inadequate, highlighting the need for multitarget modulation to enhance synergy and reduce side effects.

In this context, this paper has highlighted the multifaceted roles of natural products—derived from plants, marine organisms, and microorganisms—in modulating the complex biological events that govern wound healing. By targeting key processes such as oxidative stress, inflammation, fibroblast activity, collagen remodeling, and angiogenesis, these compounds offer a promising avenue toward regenerative outcomes that transcend traditional scarring paradigms. The historical and contemporary use of agents (such as curcumin, EGCG, asiaticoside, quercetin, and propolis—among others) demonstrates desirable effects through antioxidant, anti-inflammatory, antimicrobial, and immunomodulatory mechanisms, influencing not only the speed but also the quality of tissue regeneration. Moreover, the continuous development of effective delivery systems (e.g., hydrogels, nanocarriers, and bioactive scaffolds) has the potential to significantly enhance the bioavailability, stability, and clinical potential of these natural agents.

However, most evidence to date remains concentrated at the mechanistic (Level 3) and healing dynamic (Level 2) levels, with limited direct evaluation of clinical outcomes (Level 1) such as improved function, reduced revision rates, or cosmetically acceptable scar formation. While these bioactives show strong preclinical potential, their capacity to solve clinical problems remains unproven in most cases. Hence, the clinical use of natural-product-based products for wound healing applications necessitates a more profound mechanistic understanding and established therapy regimens.

The amalgamation of natural products with innovative biomedical technology, including nanomedicine and tissue engineering, offers a prospect to improve treatment effectiveness and connect conventional medicine with contemporary regenerative science. In addition, further research is required for optimizing compound standardization, dosing, and long-term safety, particularly in human clinical contexts. Future investigations should prioritize mechanistic studies, translational models, and controlled clinical trials to validate the therapeutic relevance of these compounds in complex wounds, including diabetic ulcers, burns, and surgical scars.

Thus, future progress will depend on multidisciplinary cooperation among pharmacologists, bioengineers, and physicians to create patient-specific, biomarker-oriented medicines. Addressing regulatory and standards obstacles will be essential for broad clinical adoption. By using advancements in customized medicine and bioprospecting, researchers may enhance therapeutic options that expedite wound healing while also improving functional and cosmetic results.

In conclusion, natural products represent a biologically rich and mechanistically diverse set of tools that may eventually support improved, patient-tailored wound care. Realizing this potential will depend on bridging the gap between molecular promise and clinical performance, a challenge that defines the next frontier in regenerative medicine.

## Figures and Tables

**Table 1:** Stages of wound healing and biophysiological events.

Phase	Cellular and Biophysiological Events	Phase Description	Refs.
Hemostasis	vascular constriction platelet aggregation, degranulation, and fibrin formation (thrombus)	initiated immediately after injury involves vasoconstriction and clot formation the clot releases cytokines and growth factors (e.g., TGF-β, PDGF, FGF, and EGF), triggering the inflammatory response	[6]
Inflammation	neutrophil infiltration monocyte infiltration and differentiation to macrophage lymphocyte infiltration	begins within 24 h and can last up to 2 weeks involves neutrophils, macrophages, and lymphocytes that clear debris and secrete cytokines (IL-1, IL-6, IL-8, and TNF-α) and growth factors to recruit repair cells	[6,7]
Proliferation	re-epithelialization angiogenesis collagen synthesis ECM formation	overlaps with inflammation characterized by epithelial cell migration, fibroblast and endothelial cell activity, angiogenesis, and ECM formation, including collagen and proteoglycans	[8]
Remodeling	collagen remodeling vascular maturation and regression	can last years involves collagen remodeling, reduced capillary density, and restoration of normal tissue structure myofibroblasts mediate wound contraction	[9,10,11]

Abbreviations: TGF-β—transforming growth factor β; PDGF—platelet-derived growth factor; FGF—fibroblast growth factor; EGF—epidermal growth factor; IL-1—interleukin-1; IL-6—interleukin-6; IL-8—interleukin-8; TNF-α—tumor necrosis factor α; ECM—extracellular matrix.

**Table 2:** Mechanisms, problems, and natural compounds/interventions related to fibrosis and wound healing.

Mechanism	Problem	Natural Compounds/Interventions	Refs.
Regulation of inflammatory cytokines	Dysregulated inflammation causes sustained expression of TNF-α, IL-6, IL-1β, promoting fibroblast proliferation and collagen synthesis.	Curcumin and green tea polyphenols reduce inflammatory cytokines, promoting regulated healing.	[10,11]
Oxidative stress inhibition	Accumulation of ROS during wound healing leads to fibroblast hyperactivation and excessive collagen production.	Antioxidants such as EGCG (green tea) and resveratrol (grapes) neutralize ROS, reducing oxidative damage and fibrosis.	[11]
Collagen accumulation control	Excessive collagen accumulation results in hypertrophic scarring through modulation of collagen synthesis and degradation.	Asiaticoside (*Centella asiatica*) and curcumin modulate MMPs and TGF-β1 expression to regulate collagen deposition and remodeling.	[6]
Angiogenesis regulation	Excessive angiogenesis can result in fibrotic tissue formation, compromising wound healing.	Polyphenols (green tea) and flavonoids (chamomile) regulate VEGF expression, facilitating optimal angiogenesis and reducing fibrosis.	[6,7]

Abbreviations: ROS—reactive oxygen species; MMPs—matrix metalloproteinases; VEGF—vascular endothelial growth factor; EGCG—epigallocatechin-3-gallate.

**Table 3:** Historical agents used to promote skin repair.

Natural Substance	Traditional Use	Claimed Healing Effects	Historical Context Notes	References
Honey	Ancient Egypt, Greece, China, WWI-era Europe (and globally)	Antimicrobial, anti-inflammatory, debriding; promotes granulation and healing	Used since antiquity (Stone Age/Egyptian tombs, ~3000 BC); Egyptians/Greeks applied honey to wounds and burns. Tested in WWI to prevent infection; recognized in Ayurvedic and Chinese tradition.	[53]
Chamomile (*Matricaria chamomilla*)	Old World (Europe, Middle East, India)	Mild anti-inflammatory, antimicrobial; soothes ulcers, cuts, and skin lesions	One of the oldest recorded medicinal herbs. Ancient Egyptians, Greeks, and Romans used chamomile poultices for inflammatory skin conditions and ulcers.	[54,55,56]
Aloe vera	Africa/India (tropical Asia, cultivated globally)	Anti-inflammatory, cooling, moisturizing; used on burns, minor cuts, skin irritations	Documented in ancient Egyptian and Ayurvedic medicine (e.g., Ebers Papyrus); “prescribed for thousands of years” for wounds and burns. Widely used in 18th–19th C. Western herbalism.	[55]
*Panax ginseng*	East Asia (China, Korea, Japan)	Adaptogenic tonic; claimed to improve circulation and tissue repair	Revered in ancient TCM and Korean medicine (e.g., Shennong’s Herbal Classic, 1st C. AD). Believed to restore vitality (“*Panax*” = “all-healing”). Modern studies note superior wound healing effects of *P. ginseng* vs. American ginseng.	[57]
*Centella asiatica* (*Gotu kola*)	South/Southeast Asia (India, Sri Lanka, China)	Stimulates collagen synthesis and angiogenesis; anti-inflammatory	Featured in Ayurvedic and Chinese pharmacopeias for centuries. Official monographs (WHO, EU) cite its wound healing and scar-reduction use. Traditional poultices of centella leaves were applied to burns and ulcers.	[58]
Turpentine oil (pine resin distillate)	Western folk medicine (Europe/N. America)	Antiseptic, counterirritant; warms tissue, relieves pain	Historical remedy (age of sail/naval medicine; 18th–19th C.) for infected wounds and skin diseases. Traditional brands mixed turpentine with lard or sugar as poultices. Modern evidence (pine resin ointment “Biopin”) shows antibacterial effects and immune modulation in wounds.	[59]

**Table 4:** Particular phytochemical compounds recognized for their role in facilitating soft tissue recovery.

Compound	Source	Biological Activity	Mechanism	Wound Healing Effects	Refs.
Curcumin	Turmeric (*Curcuma longa*)	Anti-inflammatory, antioxidant, anticoagulant	Inhibits proinflammatory NF-κB/COX-2 signaling; activates Nrf2-dependent antioxidant defence	Protects keratinocytes/fibroblasts from oxidative damage; enhances granulation and re-epithelialization in animal wounds.	[17]
Quercetin	Many fruits/vegetables (e.g., onions, apples, *Ginkgo biloba*)	Anti-inflammatory, antioxidant	Scavenges ROS; downregulates TNF-α, IL-1β, IL-6, and other cytokines	Promotes fibroblast proliferation and collagen deposition; accelerates vascularization and collagen matrix formation in wound models.	[17]
Catechins (e.g., EGCG)	Green tea (*Camellia sinensis*), cocoa	Antioxidant, antimicrobial	Potent free-radical scavengers; modulates metalloproteinases and cytokines	Reduces oxidative stress in wound bed; supports fibroblast activity and epithelial growth. Accelerates closure in treated wounds (often via catechin-enriched dressings).	[17]
Resveratrol	Grapes (skins), berries, peanuts	Anti-inflammatory, antioxidant, proangiogenic	Inhibits NF-κB and proinflammatory cytokine pathways; activates SIRT1, enhances nitric oxide/VEGF signaling	Enhances re-epithelialization and neovascularization; improves wound contraction and tensile strength (model studies). Improves diabetic wound healing via antioxidation.	[95]
Asiaticoside	*Centella asiatica* (gotu kola)	Promotes fibroblast growth and ECM synthesis	Upregulates collagen I/III and cell-cycle genes in dermal fibroblasts	Stimulates granulation tissue formation and collagen deposition. Speeds epidermal regeneration and increases tensile strength of healing skin.	[73,96]
Verbascoside (Acteoside)	*Plantago* spp. (plantain), *Lamiaceae* herbs	Anti-inflammatory, antioxidant	Inhibits proinflammatory mediators (↓TNF-α, IL-6, NO, PGE_2_)	Reduces inflammation in wound tissue; accelerates healing and re-epithelialization. (Studies show verbascoside dressings improved wound closure via reduced cytokine levels.)	[17]
Lupeol	Mango, olive, and many fruits (*Bowdichia virgilioides*, *Mangifera indica*, *Olea europaea*)	Anti-inflammatory, antioxidant	Suppresses NF-κB and IL-6; increases IL-10; upregulates angiogenic/growth factors (VEGF, EGF, TGF-β1)	Promotes angiogenesis and collagen synthesis; speeds inflammatory resolution. In animal wounds, topical lupeol creams enhance vessel growth and collagen deposition.	[17]
Gallic acid	Tea, gallnuts, berries, oak	Antioxidant, anti-inflammatory	Induces antioxidant enzymes (↑SOD2, CAT, GPx1); modulates cytokine production	Improves wound healing rate and quality. In diabetic wound models, gallic acid gels accelerated closure by boosting antioxidant defences.	[97,98]

**Table 5:** List of natural materials for ECM Restoration.

Biomaterial	Key Properties	Role in Tissue Repair	Evidence (In Vivo/Clinical)	Refs.
Collagen	Triple-helical protein Forms fibrous 3D scaffold Biocompatible Promotes cell adhesion.	Provides structural support; guides neovascularization and re-epithelialization; temporary skin substitute (especially in burns).	In animal/burn models, collagen scaffolds allow organized cell ingrowth and early wound coverage. Clinical use (dermal matrices, cultured grafts) shows improved healing in chronic ulcers.	[135]
Hyaluronic Acid (HA)	Large glycosaminoglycan Highly hydrophilic (retains water) Antioxidant (scavenges ROS). Binds CD44 on cells.	Maintains moist milieu; promotes cell migration via CD44; high-MW HA is anti-inflammatory (low-MW proinflammatory).	Topical HA enhances cell proliferation/angiogenesis in chronic wounds. In a split-thickness graft study, HA dressing accelerated closure and improved 6-month scar quality vs. control.	[135,136]
Chitosan	Cationic polysaccharide (from chitin) Biodegradable Biocompatible Hemostatic and antimicrobial.	Acts as porous 3D scaffold (e.g., electrospun or gel); promotes hemostasis, fibroblast migration, and drug delivery.	Widely studied in vitro/in vivo: chitosan dressings show reduced infection and inflammation, improved healing and collagen content. Clinical gels/gauzes are used for burns and ulcers (hemostatic and antimicrobial).	[137]
Alginate	Polysaccharide (guluronic/mannuronic acids from algae) Forms gel on wetting Absorbs ~20× its weight in exudate.	Creates moist wound environment; acts as scaffold for cell infiltration; stimulates granulation tissue and fibroblast proliferation.	Alginate dressings promote new granulation and collagen synthesis in chronic wounds. Numerous clinical trials confirm alginate sheets/foams accelerate healing of ulcers by enhancing closure rate.	[132,138]

**Table 6:** Natural compounds and their functions in promoting inflammation.

Natural Compound	Source	Anti-Inflammatory Mechanism	Effect on Wound Healing	Refs.
Epigallocatechin Gallate (EGCG)	Green tea	Inhibits NF-κB and MAPK pathways; reduces TNF-α, IL-1β, IL-6.	Promotes fibroblast and keratinocyte migration; reduces oxidative damage.	[110]
Berberine	Berberis species	Suppresses COX-2, nitric oxide, and cytokines via AMPK/NF-κB inhibition.	Improves re-epithelialization; supports healing of infected wounds.	[153]
Andrographolide	*Andrographis paniculata*	Blocks NF-κB; downregulates IL-6, TNF-α.	Accelerates inflammation resolution and granulation tissue formation.	[153]
Allicin	Garlic (*Allium sativum*)	Scavenges ROS; downregulates TNF-α, IL-1β.	Enhances macrophage activity; supports wound contraction.	[118]
Tea Tree Oil	*Melaleuca alternifolia*	Reduces IL-8 and PGE2; inhibits histamine-induced inflammation.	Reduces inflammation and infection risk in topical applications.	[119]
Flavonoids (e.g., Apigenin, Quercetin, Catechins)	Various fruits, vegetables, tea, herbs	Inhibit phosphodiesterases to delay cAMP signaling; reduce NO production; suppress IL-6 and TNF-α; modulate NF-κB activity; impair dendritic cell maturation via CD80/CD86 inhibition; target macrophage activation and differentiation.	Reduce inflammatory cell infiltration; decrease CRP levels; promote dense connective tissue formation; improve wound healing quality; show synergistic effects (e.g., quercetin + catechin) on cytokine suppression and scarring reduction.	[154]

**Table 7:** Types of wound dressings.

Type of Wound Dressing	Polymer(s)	Loaded Bioactive Agent(s)	Application/Outcome	Refs.
Films	Chitosan + Gelatin	Tannic acid + Bacterial nanocellulose	Good mechanical performance and faster full-thickness wound closure	[142,182]
Films	Chitosan	Allantoin	Excellent biocompatibility and non-toxicity with superior antibacterial efficacy	[182]
Films	Chitosan	Nanocrystalline cellulose + Calcium peroxide	Moderate WVTR and excellent cytocompatibility	[182]
Films	Chitosan	Lupeol	Initial rapid drug release followed by sustained release and good antioxidant efficacy	[182]
Films	Alginate	Hydroxyapatite	High antibacterial activity	[182]
Films	Chitosan	ZnO nanoparticles	Good antibacterial effects and increased full-thickness wound contraction rate	[142]
Films	Chitosan	Bone ash + Ciprofloxacin	Superior antimicrobial effects	[142,182]
Films	PCL	Catechin	High cell proliferation of skin cells	[182]
Films	Chitosan	-	High swelling capacity and accelerated wound healing	[142]
Films	Cellulose	-	Excellent fluid absorbing effect and fast wound closure	[182]
Membranes	CM Chitosan + HA	-	High cell viability and proliferation	[142,182]
Membranes	Chitosan	-	High growth inhibition against several bacterial strains	[182]
Membranes	Polymyxin B sulfate	Ciprofloxacin + HNTs	High swelling capacity, non-toxic, and good antibacterial activity	[182]
Films	Poly(e-caprolactone)	Usnic acid	Full-thickness wounds	[182]
Films	Chitosan/Gelatin	Silver nanoparticles/Phosphotungstic-acid–polydopamine nanoflowers	Wound healing	[142]
Films	Collagen/HPMC	Povidone–iodide	Regenerative tissue engineering	[182]
Films	Chitosan/PVA/PVP/Maltodextrin	*Satureja mutica*/*Oliveria decumbens* essential oil	Wound dressing	[182]
Films	Zein/PCL/Collagen	Zinc oxide nanoparticles + *Aloe vera*	Wound healing	[182]
Films	Collagen	Polydatin	Chronic non-healing wounds	[182]
Films	Collagen/PLA-glycolide	Glucophage	Diabetic wounds	[142,182]
Films	Sodium alginate/Gelatin	Paeoniflorin	Diabetic wounds	[142,182]
Films	Calcium alginate/PCL/Gelatin	Coconut oil	Wound healing	[182]
Films	Chitosan/Gelatin	Platelet-rich plasma	Chronic wounds	[142]
Films	Fibrin/Chitosan/Keratin	Ferulic-acid-loaded silica microspheres	Chronic and infected wounds	[142]
Films	PEG diacrylate/Catechol-HA	Ag-doped mesoporous silica nanoparticles	Wound dressing	[182]
Films	Thiolate hyaluronic acid/Silk fibroin	Bioactive glass nanoparticles	Wound healing	[142,182]
Films	Polyurethane/Chitosan	Linezolid	Diabetic wounds	[182]

**Table 8:** Smart Dressings and Injectable Hydrogels for Advanced Wound Healing Applications.

Technology Type	Formulation and Key Components	Mechanism	Stage	Reported Benefits	Ref.
Injectable Hydrogel	Crosslinked sodium alginate–poly(N-isopropylacrylamide) copolymer loaded with curcumin	Thermo-responsive (in situ gelation at ~ body temperature) Sustained antioxidant release	Preclinical	Accelerated wound contraction; significantly reduced inflammation, enhanced collagen deposition, increased fibroblast proliferation.	[192]
Smart Dressing	Self-assembling Fmoc-FFGGRGD peptide hydrogel containing resveratrol	Nanofibrous ECM-like scaffold Sustained anti-inflammatory drug release	Preclinical	Faster wound closure, well-organized collagen deposition; marked reduction of inflammatory cytokines and prevention of scar formation.	[193]
Smart Dressing	Breathable sodium alginate hydrogel incorporating curcumin and t-resveratrol	Antioxidant/antibacterial action from natural polyphenols Moisture-permeable “wet” dressing	Preclinical	Superior antioxidant and antibacterial activity; improved cell viability under oxidative stress.	[194]
Injectable Hydrogel	Hyaluronic acid gel with PCL–PEG–PCL nanomicelles loaded with curcumin	Thermo-responsive in situ gelation of HA Sustained curcumin release	Preclinical	~96% wound closure by day 14; earlier re-epithelialization, increased collagen fiber formation and enhanced angiogenesis in skin wounds.	[195]
Injectable Hydrogel	Supramolecular gelatin (host–guest) hydrogel containing resveratrol and histatin-1	Shear-thinning injectable matrix Anti-inflammatory (resveratrol) plus prohealing peptide (His-1) release	Preclinical	Promoted burn-wound healing comparable to commercial Tegaderm™; suppressed IL-6, IL-1β, TNF-α and upregulated TGF-β1/CD31 (angiogenesis).	[196]
Smart Dressing	GelMA/silk-fibroin glycidyl-methacrylate hydrogel with mesoporous silica NPs loaded with resveratrol and platelet-derived vesicles	Photo-crosslinked matrix Sustained dual delivery of anti-inflammatory resveratrol (in MSNs) and proangiogenic EVs	Preclinical	In diabetic wounds: reduced TNF-α, iNOS; increased anti-inflammatory TGF-β1, Arg-1; enhanced angiogenesis and accelerated healing.	[197]
Smart Dressing	Gelatin-methacryloyl (GelMA)/ionic-liquid hydrogel loaded with resveratrol	Photo-crosslinked Ionic liquid imparts intrinsic antibacterial activity pH-neutral sustained release	Preclinical	Accelerated diabetic wound closure with reduced inflammation and robust neovascularization; activated proangiogenic PI3K/AKT pathways.	[198]
Injectable Hydrogel	Thermosensitive chitosan/ε-polylysine hydrogel integrated with Cu/Mg bimetallic nanoenzymes	Thermo-responsive gelation ROS-scavenging via Cu/Mg-MOF nanozymes	Preclinical	~90.6% wound closure by day 14 (vs. 55.4% untreated); enhanced collagen deposition, re-epithelialization, angiogenesis, and immunomodulation in diabetic wounds.	[199]

**Table 9:** Summary of clinical trials evaluating natural-product-based therapies for wound healing.

Natural Product or Formulation	Study Design and Patient Population	Outcome Measures	Main Findings	Ref.
Centella/Echinacea/Sambucus patch (PerioPatch^®^)	RCT in rats (gingival incisions, n = 48)	Epithelial gap; collagen content; cell proliferation	Herbal-patch group had smaller epithelial gaps and higher collagen content and cell proliferation versus placebo.	[200]
Papaya (*Vasconcellea*) latex protease (P1G10) ointment	Double-blind RCT, n = 50 (neuropathic diabetic foot ulcer patients)	% of wounds with 100% and ≥80% closure; healing time	More patients healed with P1G10 (11/50 full closures vs. 5/50 controls) and higher ≥80% closures; healing occurred in a shorter time with no adverse effects.	[217]
*Calendula officinalis* hydroglycolic extract	Prospective open-label pilot, n = 41 (diabetic foot ulcer patients)	% wound closure; time to heal; bacterial load; pain	78% of ulcers achieved complete closure by 30 weeks (mean ~15.5 weeks); bacterial colonization and patient pain were reduced after treatment.	[218]
North Atlantic cod fish skin graft	Multinational open-label RCT, n = 255 (deep diabetic foot ulcers)	% healed at 16/24 weeks; time to healing	44% healed at 16 weeks with fish skin vs. 26% with standard care (*p* < 0.001); at 24 weeks 55% vs. 38%. Fish skin treatment accelerated healing (hazard ratio ~1.59).	[204]
*Mimosa tenuiflora* (*tepezcohuite*) bark hydrogel	RCT, n = 41 (venous leg ulcer patients; extract gel vs. hydrogel)	Ulcer area reduction; re-epithelialization	Both groups showed similar ulcer area reduction; the mimosa extract gel was *not superior* to placebo hydrogel (no significant differences).	[219]
Combined herbal ointments (Plantoderm^®^ + Fitoven^®^)	Pilot RCT, n = 34 (17 combined herb vs. 17 standard care, venous ulcers)	Ulcer area reduction; bacterial load	Herbal-treatment group saw ~42.7% ulcer area reduction vs. ~35.6% in controls at 7 weeks; also greater bacterial clearance (four ulcers became culture-negative).	[211]
*Aloe vera* gel	RCT, n = 68 (first/second-degree burns; 34 aloe vs. 34 silver sulfadiazine)	Time to epithelialization; pain; itch	Both groups fully epithelialized by ~2 weeks, but the aloe group healed faster and had significantly less pain and itch, especially noticeable by day 7.	[211]
Glycerol-preserved tilapia fish skin dressing	Phase III RCT, n = 115 (partial-thickness burns; tilapia vs. SSD)	Re-epithelialization time; dressing changes; pain; cost	Tilapia grafts accelerated wound closure (mean ~9.7 days vs. 10.2 days with SSD, *p* = 0.001). Patients needed fewer dressing changes, reported lower pain, and treatment cost was ~42% lower.	[217]
Lavender (*Lavandula*) essential oil (sitz bath/spray)	Systematic review of 5 RCTs (postpartum episiotomy wounds)	Wound healing (REEDA score); pain score	Lavender baths significantly sped wound closure and improved REEDA scores versus controls and reduced perineal pain in all trials (no reported adverse effects).	[215]
